# Tau protein aggregation is associated with cellular senescence in the brain

**DOI:** 10.1111/acel.12840

**Published:** 2018-10-11

**Authors:** Nicolas Musi, Joseph M. Valentine, Kathryn R. Sickora, Eric Baeuerle, Cody S. Thompson, Qiang Shen, Miranda E. Orr

**Affiliations:** ^1^ Barshop Institute for Longevity and Aging Studies University of Texas Health Science Center at San Antonio San Antonio Texas; ^2^ San Antonio Geriatric Research Education and Clinical Center South Texas Veterans Health Care System San Antonio Texas; ^3^ Glenn Biggs Institute for Alzheimer’s & Neurodegenerative Diseases San Antonio Texas; ^4^ Research Imaging Institute University of Texas Health Science Center San Antonio San Antonio Texas

**Keywords:** aging, Alzheimer’s disease, cellular senescence, neurodegeneration, senolytic, tau

## Abstract

Tau protein accumulation is the most common pathology among degenerative brain diseases, including Alzheimer's disease (AD), progressive supranuclear palsy (PSP), traumatic brain injury (TBI), and over twenty others. Tau‐containing neurofibrillary tangle (NFT) accumulation is the closest correlate with cognitive decline and cell loss (Arriagada, Growdon, Hedley‐Whyte, & Hyman, 1992), yet mechanisms mediating tau toxicity are poorly understood. NFT formation does not induce apoptosis (de Calignon, Spires‐Jones, Pitstick, Carlson, & Hyman, 2009), which suggests that secondary mechanisms are driving toxicity. Transcriptomic analyses of NFT‐containing neurons microdissected from postmortem AD brain revealed an expression profile consistent with cellular senescence. This complex stress response induces aberrant cell cycle activity, adaptations to maintain survival, cellular remodeling, and metabolic dysfunction. Using four AD transgenic mouse models, we found that NFTs, but not Aβ plaques, display a senescence‐like phenotype. *Cdkn2a* transcript level, a hallmark measure of senescence, directly correlated with brain atrophy and NFT burden in mice. This relationship extended to postmortem brain tissue from humans with PSP to indicate a phenomenon common to tau toxicity. Tau transgenic mice with late‐stage pathology were treated with senolytics to remove senescent cells. Despite the advanced age and disease progression, MRI brain imaging and histopathological analyses indicated a reduction in total NFT density, neuron loss, and ventricular enlargement. Collectively, these findings indicate a strong association between the presence of NFTs and cellular senescence in the brain, which contributes to neurodegeneration. Given the prevalence of tau protein deposition among neurodegenerative diseases, these findings have broad implications for understanding, and potentially treating, dozens of brain diseases.

## 1 INTRODUCTION

The underlying processes driving chronic neurodegeneration in Alzheimer’s disease (AD) and related neurodegenerative disorders are largely unknown, and disease‐modifying treatments remain elusive. The accumulation of tau protein is the most common pathology among these diseases making tau an appealing molecular target for intervention (Orr, Sullivan, & Frost, 2017). Tau‐containing neurofibrillary tangles (NFTs) closely track with disease severity in human AD (Arriagada, Growdon, Hedley‐Whyte, & Hyman, 1992); however, NFT‐containing neurons are long‐lived and do not induce immediate cell death (de Calignon, Spires‐Jones, Pitstick, Carlson, & Hyman, 2009). *In silico* modeling predicts that NFT‐containing neurons may survive decades (Morsch, Simon, & Coleman, 1999), which suggests that non‐cell autonomous mechanisms may contribute to NFT‐associated toxicity.

The experimental data from various studies indicate that tau pathology may be associated with cellular senescence. This complex stress response induces a near permanent cell cycle arrest, adaptations to maintain survival, cellular remodeling, metabolic dysfunction, and disruption of surrounding tissue due to the secretion of toxic molecules (Childs et al., 2016). While many of these features have been described in AD brains and transgenic animal models throughout the literature (e.g., aberrant cell cycle activity, p16^INK4A^ co‐localization with NFTs (Arendt, Rodel, Gartner, & Holzer, 1996), decreased lamin B1, and heterochromatin relaxation (Frost, Bardai, & Feany, 2016); a role for cellular senescence in AD‐associated neurodegeneration has not been investigated. We hypothesized that tau accumulation may activate this stress response and thereby initiate a chronic degenerative process culminating in neuron loss and brain dysfunction. To test this hypothesis, we examined human brain tissue with NFT pathology and utilized AD transgenic mouse models that develop tau‐associated pathologies. Also, we employed methods to genetically reduce NFTs and pharmacologically clear senescent cells. Our results indicate that NFTs induce cellular senescence in transgenic mice and postmortem human brain tissue. We also found that senolytics decreased cortical NFT burden, brain atrophy, and neuron loss in an advanced age (20 months old) transgenic mouse model of tau‐associated neurodegeneration.

## 2 RESULTS

### 2.1 NFT‐bearing neurons from postmortem AD brain tissue displayed a senescence‐like transcriptomic profile

**Figure 1:**
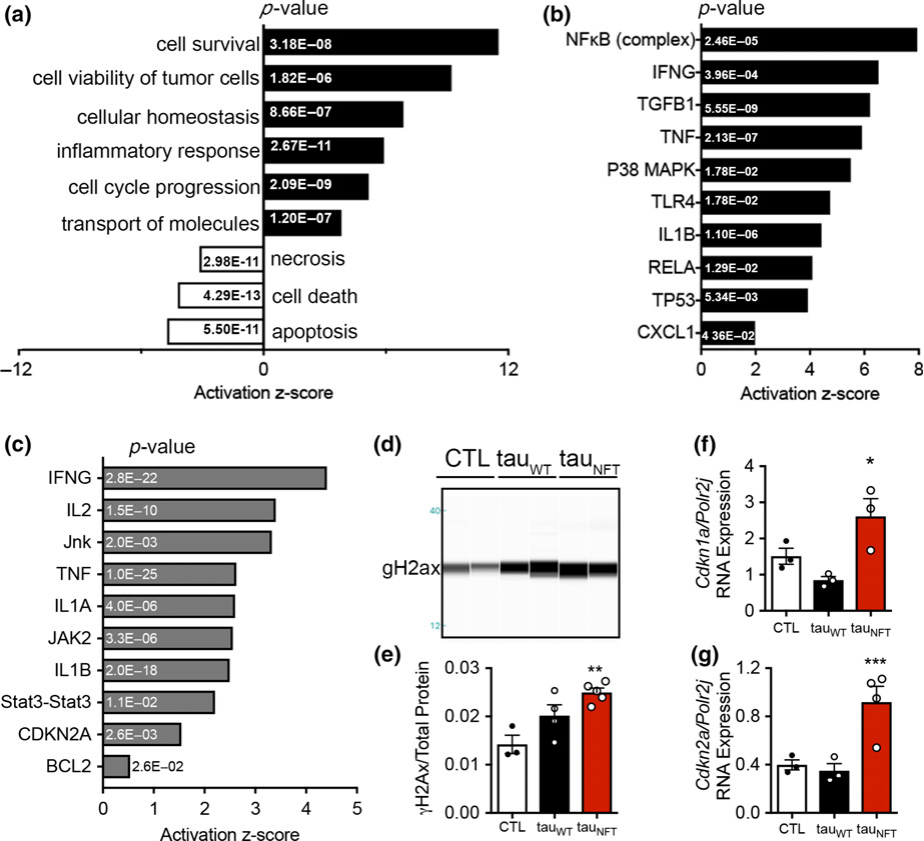
Neurofibrillary tangles were associated with cellular senescence‐associated gene pathways in human Alzheimer’s disease neurons and tau transgenic mouse brains*. *(a) Pathways and predicted upstream regulators identified by ingenuity pathway analyses (IPA, QIAGEN) as significantly enriched in Alzheimer’s disease patient‐derived neurons with neurofibrillary tangles compared to non‐tangle‐containing neurons; *z*‐score plotted on *x*‐axis and (*p*‐value) indicated in bar graph. Cellular functions and (b) predicted upstream regulators employed by neurofibrillary tangle‐containing neurons derived from Alzheimer’s disease patient are shown. (c) Predicted upstream regulators of gene transcription in tau_NFT_ mice after the onset of neurofibrillary tangles (~6 months old vs. ~2 months old); *z*‐score plotted on *x*‐axis and (*p*‐value) indicated in bar graph. (d‐e) Representative immunoblot generated by capillary electrophoresis on chromatin‐bound fractions from mouse forebrain homogenate probed with anti‐γ‐H2ax antibody. (e) Densitometric normalization of γ‐H2ax to total protein content (CTL: *n* = 3; tau_WT_ *n* = 4; tau_NFT_: *n* = 5; ANOVA, *p = *0.0056. Mice aged 16 to 18 months old). (f–g) Quantitative gene expression on RNA isolated from CTL (open bar, *n* = 3), tau_WT_ (closed bar, *n* = 3), and tau_NFT_ (red bar, *n* = 4) mouse forebrain targeting (f): *Cdkn2a, p = *0.0066, *and *(g) *Cdkn1a, p = *0.0207. Gene expression was analyzed by one‐way ANOVA Tukey’s multiple comparison *post hoc*. Data are graphically represented as mean ± *SEM*

We queried the publicly available GEO Profiles database (Barrett et al., 2013) for gene sets specific to NFTs. We evaluated laser capture‐microdissected cortical neurons containing NFTs from AD brains (GEO accession GDS2795) and compared them to adjacent histopathologically normal neurons for a within‐subject study design (Dunckley et al., 2006). NFT‐containing neurons upregulated genes involved in cell survival and viability, inflammation, cell cycle progression and molecular transport and downregulated apoptosis, necrosis, and cell death pathways (Figure 1a). NFκB, a pro‐survival master transcriptional regulator of inflammation, was the highest predicted upstream regulator of the NFT gene expression profile. In agreement with inflammatory activation, other predicted upstream regulators included IFNG, TNF, TLR4, IL1B, and CXCL1 (Figure 1b). Collectively, the molecular pathways identified in the NFT analyses resembled cellular senescence.

### 2.2 NFTs were associated with a senescence‐associated transcriptomic profile in tau transgenic mice

We used the rTg(tauP301L)4510 transgenic mouse line, hereon referred to as “tau_NFT_” to investigate a link between NFT formation and a senescence‐like phenomenon in neurodegeneration. These mice develop well‐characterized, aggressive, tau pathology in forebrain regions concomitant with neurodegeneration and cognitive deficits (Santacruz et al., 2005; pathology illustrated in Supporting Information Figure S1). Mice that overexpress wild‐type human tau, “tau_WT_,” express the same level of transgenic human tau protein as tau_NFT_, but acquire age‐dependent tau pathogenesis at a much slower rate and are used to identify effects of elevated pre‐pathogenic tau (Hoover et al., 2010; Supporting Information Figures S1 and S2); age‐matched tau_NFT_ littermate mice without human tau overexpression serve as wild‐type controls, “CTL”. To determine whether NFT‐containing neurons in mice induced a gene expression profile resembling cellular senescence, we assessed hippocampal gene expression patterns in tau_NFT_ mice before (~2 months old) and after (~6 months old) NFT formation (GSE56772). Consistent with NFTs from human AD, mouse NFTs also caused significant activation scores for IFNG, TNF, and IL‐1B, as well as enrichment in other senescence‐associated JAK, STAT, CDKN2A, and BCL2 predicted upstream regulators (Figure 1c) indicating translational relevance for using tau_NFT_ mice to explore our hypothesis.

### 2.3 Evidence of DNA damage, SASP, and NFκB activation were associated with NFTs

Senescence‐inducing stressors often inflict DNA damage that drives production of the SASP (Rodier et al., 2009). Tau_NFT_ mouse brains displayed significantly elevated histone γ‐H2ax, a sensitive marker of both double‐stranded DNA breaks and cellular senescence (Sedelnikova et al., 2004; *p* = 0.0056; Figure 1d–e). The cell cycle protein p21, encoded by *Cdkn1a*, is upregulated in many senescent cell types and has been associated with DNA damage during neuronal aging (Jurk et al., 2012). Similarly, elevated expression of the cyclin‐dependent kinase inhibitor 2a, *Cdkn2a*, is one of the most robust markers of cellular senescence, and its protein product, p16^INK4A^, colocalizes with NFTs in human AD (Arendt et al., 1996). Because anti‐p21 and anti‐p16^INK4A^ antibodies are notoriously poor in mouse tissue, we exclusively measured *Cdkn1a* and *Cdkn2a* gene expression. Tau_NFT_ brains expressed three‐fold higher *Cdkn1a* than control mice (*p* = 0.0178, Figure 1f), which was replicated in a separate mouse cohort (*p* = 0.0086, Supporting Information Figure S2f). Moreover, *Cdkn2a* was expressed at levels 2.7‐ and 2.6‐fold higher in tau_NFT_ than CTL and tau_WT_, respectively (*p* = 0.0303 and *p* = 0.0352, respectively; Figure 1g); this effect was replicated in an independent mouse cohort (*p* = 0.0016, Supporting Information Figure S2g).

**Figure 2:**
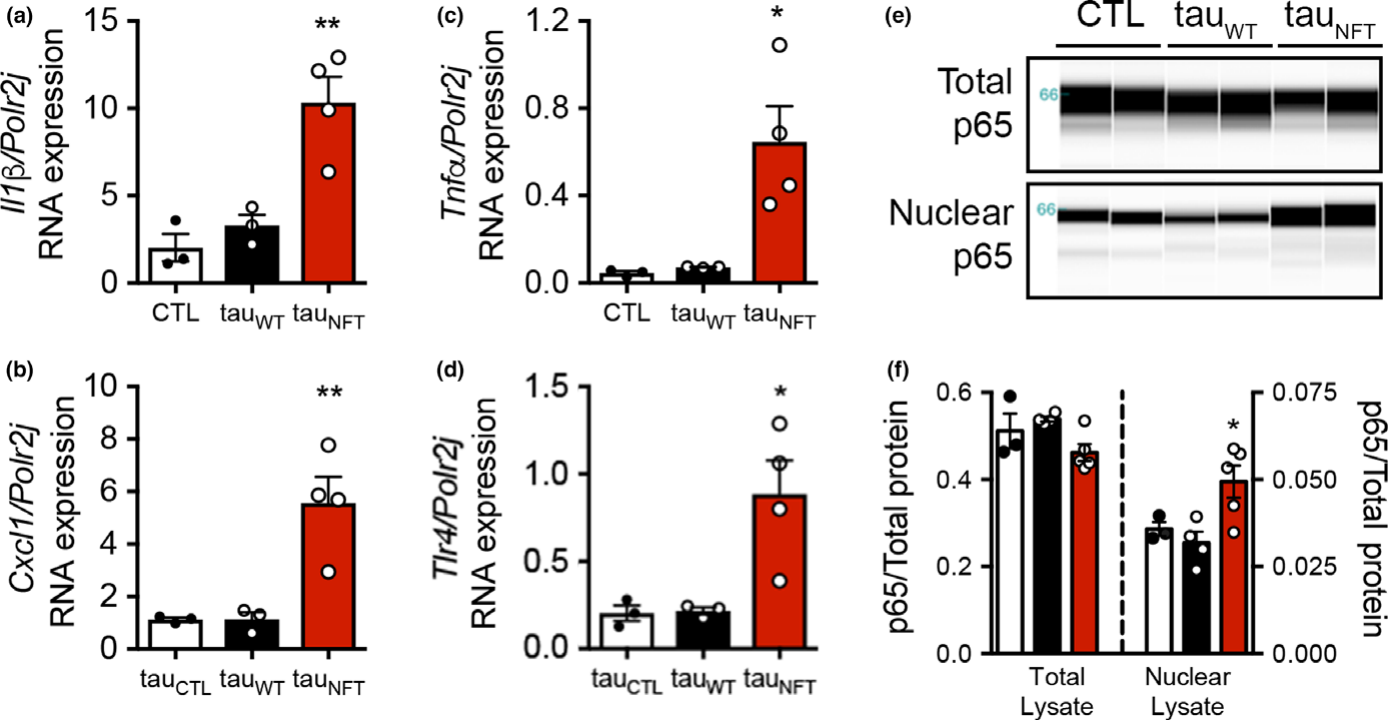
Neurofibrillary tangles were associated with upregulation of SASP gene expression and NFκB activation*. *(a) Quantitative gene expression on RNA isolated from CTL (open bar, *n* = 3), tau_WT_ (closed bar, *n* = 3), and tau_NFT_ (red bar, *n* = 4) mouse forebrain targeting SASP‐associated genes *Il1b, p = *0.0025; (b) *Cxcl1, p = *0.0040; (c) *Tnfa*, *p = *0.0114; and (d) *Tlr4*, *p = *0.0144. (d) Immunoblot generated by capillary electrophoresis on subcellular fractionated mouse forebrain homogenate probed with anti‐NFκB p65 antibody. Total cellular p65 (top blot) and nuclear‐localized p65 protein levels (bottom blot) were (e) normalized to total protein content. Total p65, *p* = 0.0758; nuclear p65, *p* = 0.0223. CTL: open bar, *n* = 3; tau_WT_: closed bar, *n* = 4; tau_NFT_: red bar, *n* = 5. In all experiments, mice were aged 16–18 months old; both males and females were included. Significance was determined by one‐way ANOVA Tukey’s multiple comparison *post hoc*. Data are graphically represented as mean ± *SEM*

Senescent cells exert chronic tissue degeneration through secretion of toxic SASP (Coppe et al., 2010). Consistent with the transcriptomic profile in human NFT‐bearing neurons and mouse brain tissue (Figure 1a‐c), SASP genes were found to be upregulated in tau_NFT_ brains, that is, *Il1b* was four‐ and twofold higher than CTL and tau_WT_, respectively; and *Cxcl1* was fourfold higher than both control genotypes; *Tnfa* was 13‐ and eightfold higher than CTL and tau_WT_, respectively; *Tlr4* was threefold higher than both control genotypes (Figure 2a‐d). Further gene expression analyses allowed us to define an array specific to tau pathology in tau_NFT_ brains (Supporting Information Figure S2e). NFκB regulates the pro‐survival, pro‐inflammatory SASP gene expression profile characteristic of cellular senescence (Salminen & Kaarniranta, 2011). Consistent with NFκB pathway activation and the SASP profile, nuclear‐localized NFκB p65 was significantly increased in tau_NFT_ brains (Figure 2e‐f). In all measures, tau_WT_ mice were not significantly different from CTL. These results suggest that insoluble tau and/or post‐translational modifications associated with insoluble tau, but not general tau overexpression, were responsible for the senescence‐associated profile (i.e., DNA damage, NFκB activation, and upregulated SASP; Figures 1, 2 and Supporting Information Figure S2).

### 2.4 SA β‐gal activity did not correlate with NFTs or brain atrophy

In regenerative tissues and *in vitro* cultures, senescent cells may exhibit SA β‐gal activity, which is a measure of lysosomal galactosidase activity at pH 6.0 and indicative of altered/expanded lysosomal compartments (Severino, Allen, Balin, Balin, & Cristofalo, 2000). The examination of the gene that codes for the hydrolase enzyme, galactosidase beta (β) 1 (*Glb1*), revealed that tau_NFT_ mice expressed higher *Glb1* gene expression than controls (Supporting Information Figure S3). However, staining for β‐gal hydrolase activity at pH 6.0 revealed fewer positive cells than controls. Furthermore, SA β‐gal‐reactive cells were observed even in very young mice (1 month old) and the number of SA β‐gal‐reactive cells was positively correlated with brain mass (*R* ^2^ = 0.4852, *p* = 0.0039 Supporting Information Figure S3). While our results indicate that SA β‐gal reactivity did not correlate with other senescence markers or brain atrophy, the observed increase in *Glb1* gene expression along with a decrease in lysosomal activity at pH 6.0, compared to controls, is suggestive of tau‐associated lysosomal defects, which have been reported by others (Caballero et al., 2018; Wang, Martinez‐Vicente, et al., 2009).

### 2.5 NFT‐containing brain tissue displayed aberrant cellular bioenergetics

**Figure 3:**
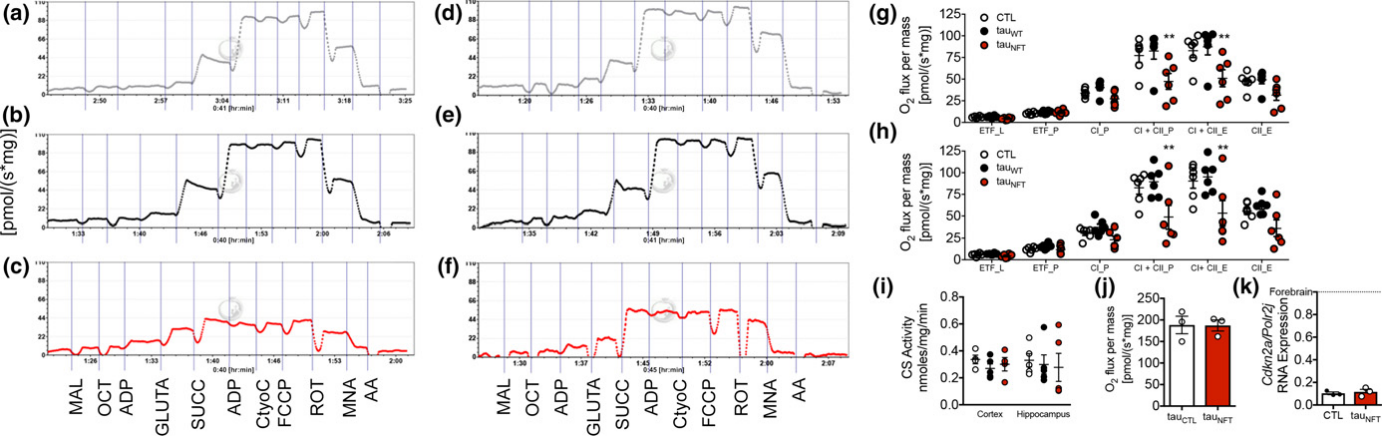
Brain regions with neurofibrillary tangles displayed altered cellular respiration. (a–c) Representative respirometric traces from cortical and (d–f) hippocampal tissues using the SUIT protocol to measure oxygen consumption (top gray traces: CTL; black middle traces: tau_WT_; bottom red traces: tau_NFT_). (g) Tissue mass‐specific respiration analyses in cortical and (h) hippocampal tissue. Two‐way ANOVA Tukey’s multiple comparison *post hoc*: **p < 0.005. (i) Biochemical analyses of citrate synthase (CS) activity to assess total mitochondrial content in the cortex and hippocampus (*n* = 5/group). Experimental mice were aged 16–18 months old with *n* = 6/group; both males and females were included. (j) Total oxygen consumption and (k) *Cdkn2a *gene expression were measured in the cerebellum, a brain region devoid of NFTs. *n* = 3/group. Data are graphically represented as mean ± *SEM*. ETF_L (fat oxidation in the absence of ADP [state 2]), ETF_P (fat oxidation coupled to ATP production), CI_P (complex I activity linked to ATP production [state 3]), CI + CII_P (complex I and complex II linked respiration [state 3]), CI + CII_E (complex I and complex II linked respiration uncoupled [maximum respiration]), and CII_E (complex II activity uncoupled). Data are graphically represented as mean ± *SEM*

Mitochondrial dysfunction is obligatory for SASP production and cellular senescence (Correia‐Melo et al., 2016; Hutter et al., 2004). To examine mitochondrial bioenergetics, we performed high‐resolution respirometry to yield accurate quantitative measurements of oxidative phosphorylation in response to specific substrates for complex I, complex II, fat oxidation, and electron‐transfer system (ETS) capacity. Across genotypes, we compared cortex, hippocampus, and cerebellum. This allowed for the evaluation of specific differences in oxygen consumption due to elevated transgenic tau (comparing CTL with tau_wt_ and tau_NFT_), pathogenic tau‐specific effects (comparing tau_wt _to tau_NFT_), as well as the interaction among brains regions and tau expression (e.g., cortex and hippocampus express transgenic tau and develop NFTs, but cerebellum does not). We found a significant genotype main effect for oxygen flux in both cortex and hippocampus, indicating that global respiratory capacity was impaired in NFT‐containing brain regions (*p* < 0.0001; Figure 3), an effect primarily driven by CI + CII respiration coupled to ATP production (cortex: *p* = 0.0034; hippocampus: *p* = 0.0215; Figure 3g,h, respectively), and uncoupled or maximum respiratory capacity (cortex: *p* = 0.0248; hippocampus: *p* = 0.0261; Figure 3g,h, respectively). These changes were different between tau_NFT_ and each of the control mouse lines, CTL, and tau_WT _mice. Because tau_WT_ and tau_NFT_ mice express comparable total tau levels, alterations to respiratory capacity cannot be attributed to tau overexpression. Citrate synthase activity is a surrogate marker of total mitochondrial content/mass and was similar across genotypes and brain regions (Figure 3i) suggesting that the defects in cellular respiration were due to altered mitochondrial quality and not content/mass. Moreover, tau_NFT_ cerebellum did not show deficits in cellular respiration or *Cdkn2a* upregulation (Figure 3j,k), indicating that senescence‐associated mitochondrial dysfunction was present only in brain regions with persistent pathogenic tau expression.

### 2.6 *Cdkn2a* upregulation occurred with NFT onset and correlated with NFT density

**Figure 4:**
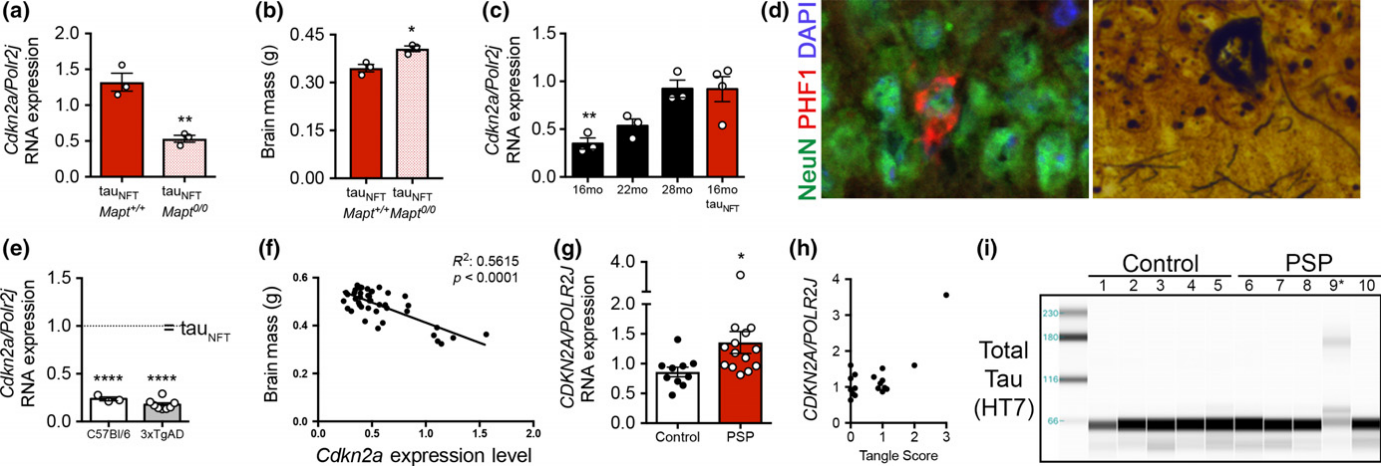
Senescence‐associated *Cdkn2a* was significantly upregulated in mouse and human brains with neurofibrillary tangles and tracked with total tangle deposition and brain atrophy*. *(a) Genetically ablating endogenous mouse tau to significantly reduce neurofibrillary tangle load resulted in a concomitant 60% reduction in *Cdk2na *expression (two‐tailed *t* test: *p = *0.0041; *n* = 3/group) and (b) significant reduction in brain atrophy (two‐tailed *t* test, *p = *0.0143; *n* = 3/group). (c) Tracking *Cdkn2a *expression in tau_WT_ mice revealed a significant age‐dependent increase (one‐way ANOVA: *p* = 0.0043; *n* = 3/group for tau_WT_ and *n* = 4 tau_P301L_). In contrast to significantly lower expression than tau_NFT_ mice at 16 months old (*p = *0.0075), Dunnett's multiple comparison test indicated that at 22 months of age, tau_WT_ mouse *Cdkn2a *expression was no longer statistically lower than tau_NFT_ mice (*p* = 0.0577) and by 28–30 months they were are statistically the same (*p* = 0.999). (d) Immunofluorescence and Bielschowsky silver staining revealed neurofibrillary tangles in 18‐month‐old tau_WT_ mouse hippocampal CA1 (NeuN, neuron, green; PHF1: phosphorylated tau, red; DAPI, blue, nuclei). (e) qPCR analyses of RNA extracted from 3xTgAD mice with Aβ plaques were compared to tau_NFT_ set at *y* = 1. 3xTgAD *Cdkn2a *expression was no different than age‐matched C57BL/6 mice (two‐tailed *t* test, *p = *0.1081; *n* = 3 WT, *n* = 6 3xTgAD; *n* = 4 tau_NFT_). Both mouse cohorts expressed significantly less *Cdkn2a *than tau_NFT_ mice (one‐way ANOVA: *p* < 0.0001). (f) *Cdkn2a* expression level was significantly correlated with brain atrophy (*R* ^2^ = 0.5615, *p* < 0.0001; *n* = 43). (g) qPCR analyses of RNA extracted from brains from control older adult humans (*n* = 10; ave. age = 85.70 years) and age‐matched progressive supranuclear palsy (*n* = 14; ave. age = 83.86 years) indicated a 57% upregulation of *CDKN2A* with progressive supranuclear palsy diagnosis (unpaired *t* test, *p* = 0.0415) that (h) positively correlated with neurofibrillary tangle deposition in the parietal lobe (ANOVA, *p* = 0.0008; Kendall’s Tau rank correlation *p* = 0.059). (i) Immunoblot generated by capillary electrophoresis on cortical brain homogenate from control and progressive supranuclear palsy human brains probed with total tau antibody, HT7. The individual with the highest *CDKN2A* expression (panel g) displayed high molecular weight tau, lane 9*. Data are graphically represented as error bars, mean ± *SEM*

We pursued multiple genetic approaches to determine whether senescence was mechanistically linked to NFT density, NFT onset, or merely protein accumulation. Reducing NFT load in age‐matched animals is not feasible; once NFTs form, they cannot be therapeutically eliminated. However, genetically ablating endogenous mouse tau (microtubule‐associated protein tau, *Mapt*) reduces NFT pathology and neurodegeneration in tau_NFT_ mice (tau_NFT_‐*Mapt^0/0^*; Wegmann et al., 2015). The reduced tau pathology corresponded with 60% lower *Cdkn2a* expression (*p* = 0.0041, Figure 4a), decreased SASP (Supporting Information Figure S4), and decreased brain atrophy (tau_NFT_‐*Mapt^0/0^*: 0.4058 ± 0.009 vs. age‐matched tau_NFT_‐*Mapt^wt/wt^*: 0.3451 ± 0.0116; 17.5% difference, *p* = 0.0143, Figure 4b). Tau_NFT_ mice develop aggressive tauopathy with NFT formation in early life and show a senescence‐associated transcriptomic profile with NFT onset (Figure 1c). To detect subtle cellular changes associated with different stages of age‐associated NFT development and progression, we focused on tau_WT_ mice between 16 and 28 months old. *Cdkn2a* gene expression increased significantly during this age interval, and at 28 months of age, tau_WT_ *Cdkn2a* expression was similar to that of 16‐month‐old tau_NFT_ mice (Figure 4c). Concomitantly, at this age, tau_WT _mice developed NFTs as visualized by Bielschowsky silver staining and immunofluorescence analyses (Figure 4d). These results provide additional evidence for the association between NFT formation and senescence‐associated *Cdkn2a* upregulation.

### 2.7 *Cdkn2a* upregulation was specific to NFT tau pathology and correlated with brain atrophy

To determine whether *Cdkn2a* expression was driven specifically by NFTs, or whether AD‐associated Aβ protein deposition also increased *Cdkn2a*, we utilized 3xTgAD mice that acquire both AD‐associated pathologies with Aβ deposition and NFT onset at 6 and 18 months of age, respectively (Oddo et al., 2003). In 15‐month‐old mice with heavy Aβ deposition and phosphorylated tau, but lacking NFT pathology (Orr, Salinas, Buffenstein, & Oddo, 2014), *Cdkn2a* expression was not elevated (Figure 4e). These data indicate that *Cdkn2a* expression was neither a response to general protein accumulation, nor to pre‐NFT tau pathology, but instead required the presence of NFTs. Further, when plotted against brain weight, *Cdkn2a* expression was a strong predictor of brain atrophy across mouse lines (*p* < 0.0001, *R* ^2^ = 0.5615; Figure 4f).

### 2.8 *CDKN2A* was upregulated in NFT‐containing brains from patients with progressive supranuclear palsy

Tau pathology is common among >20 brain diseases. To investigate whether the findings in human AD neurons and transgenic mice translated to human brains with pure tauopathy (i.e., NFT pathology without other protein aggregates such as Aβ), we acquired human brain tissue with histopathologically confirmed progressive supranuclear palsy (PSP; Table 1 for patient characteristics). PSP is an age‐associated tauopathy that clinically manifests as parkinsonism with additional motor abnormalities and cognitive dysfunction (Orr et al., 2017) and is neuropathologically defined by the accumulation of four‐repeat (4R) tau, NFTs, gliosis, and neurodegeneration (Flament, Delacourte, Verny, Hauw, & Javoy‐Agid, 1991). Consistent with the results from transgenic mice, *CDKN2A* was upregulated in PSP brains (*p* = 0.0415, Figure 4g) and expression correlated with NFT deposition, specifically in the parietal lobe (ANOVA, *p* = 0.0008; Kendall's Tau rank correlation, *p* = 0.059, Figure 4h). Moreover, one individual with the worst cognitive performance, Mini–Mental State Examination (MMSE) score of 12, displayed the highest level of *CDKN2A* expression, and high molecular weight tau (Figure 4i). Collectively, these findings led us to conclude that NFTs were directly linked to senescence‐associated *Cdkn2a* upregulation, which in turn was a strong predictor of neurodegeneration and cognitive decline.

**Table 1:** Human postmortem brain characteristics

	Control (*n* = 10)	PSP (*n* = 14)	*p*‐Value
Age at death (years)	85.70 ± 2.81	83.86 ± 3.08	0.6765
Sex (M/F)	6/4	9/5	N/A
Last MMSE score	27.67 ± 0.87 (*n* = 9)	21.00 ± 2.02	0.0194
Brain mass (g)	1,169 ± 17.83	1,139 ± 43.21	0.5800
Total tangles	4.03 ± 0.77	7.64 ± 0.66	0.0017

### 2.9 Senolytic treatment reduced NFT burden and neurodegeneration

**Figure 5:**
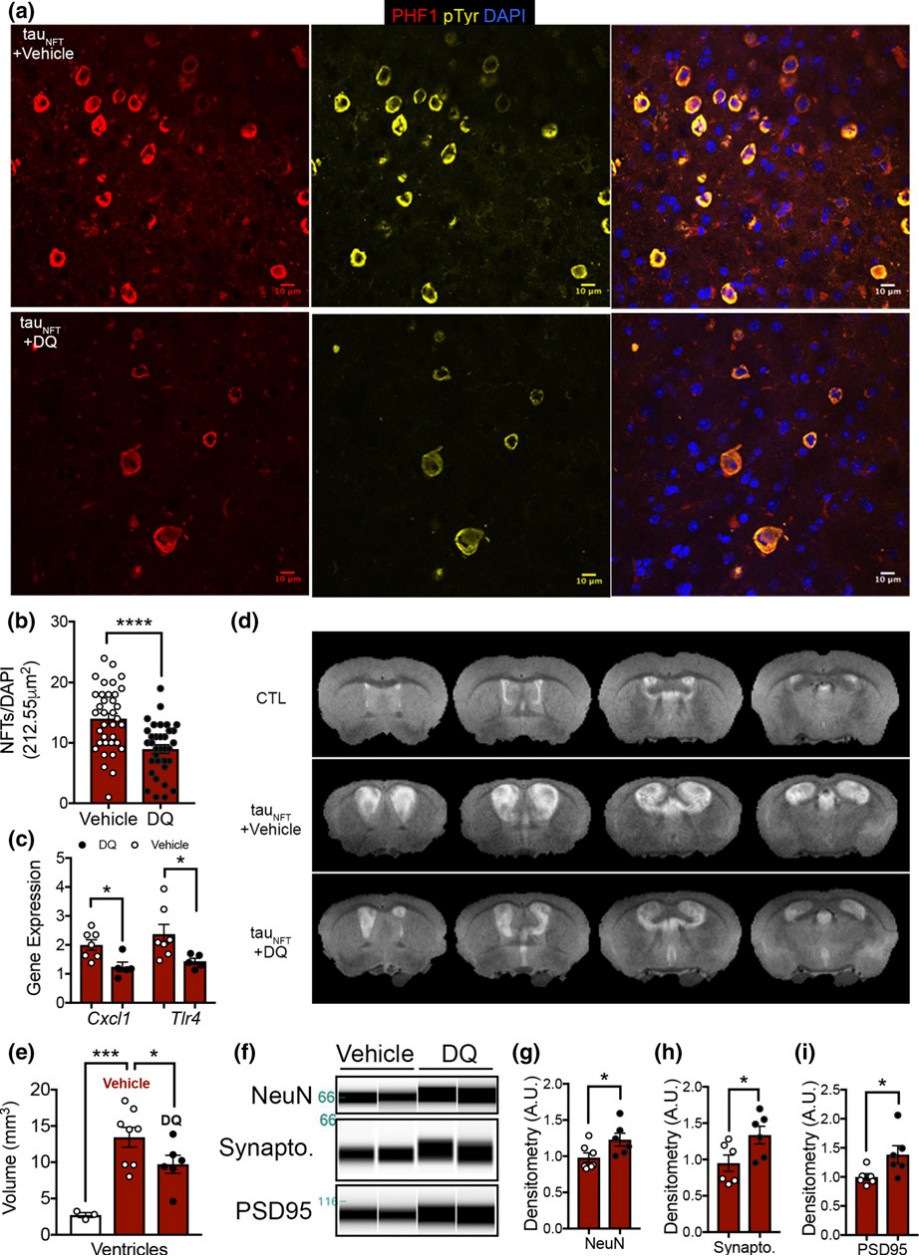
Senolytic treatment reduced neurofibrillary tangle burden, ventricular enlargement, and neurodegeneration in 23‐month‐old tau transgenic mice. (a) Representative brain images analyzed for neurofibrillary tangles in tau transgenic mice treated with either vehicle or dasatinib and quercetin (DQ). (phosphorylated tau, PHF1, red; total tyrosine phosphorylation, pTyr, yellow; and DAPI nuclei; blue. Scale bar = 10 µm). (b) Neurofibrillary tangle counts from *n* = 3 mice/group sampled from 12 cortical images/mouse and analyzed with unpaired two‐tailed *t* test, ****p < 0.0001. (c) DQ significantly reduced hippocampal *Tlr4*, *p* = 0.0459, and *Cxcl1*, *p* = 0.0142, gene expression as measured by qPCR. Vehicle‐treated (open symbols, *n* = 7) and DQ‐treated (red closed symbols, *n* = 5); data analyzed by unpaired two‐tailed *t* test. (d) Representative brain images from anatomical T2‐weighted MRI. (e) Quantification of ventricle volume analyzed by one‐way ANOVA, *p* = 0.0010; Holm–Sikak's post hoc: ***p = 0.0007, *p = 0.05. (f) Immunoblot generated by capillary electrophoresis on forebrain homogenates (*n* = 6/group) with antibodies against neuronal proteins NeuN, synaptophysin (synapto.), and PSD95 normalized to total protein. (g) Neuronal protein expression was normalized to total protein and analyzed by unpaired two‐tailed *t* test, NeuN: *p = 0.0432, (h) synaptophysin (synapto): *p = 0.0416 and (i) PSD95: *p = 0.0398. *n* = 6/group. Data represented as mean ± *SEM*

Senescent cells comprise a small proportion of total cellular makeup within a tissue (~15%; Herbig, Ferreira, Condel, Carey, & Sedivy, 2006). Nonetheless, genetically (Baker et al., 2011) or pharmacologically (Zhu et al., 2015) clearing even a small percentage of these cells improves health span and delays age‐associated diseases (Kirkland et al., 2017). We used some of the best characterized senolytics to date, dasatinib and quercetin (DQ), to determine the utility of targeting cellular senescence to treat tau‐associated neurodegeneration in late life. Beginning at 20 months old, tau_NFT_‐*Mapt^0/0^* and nontransgenic *Mapt^0/0^* mice were randomized to receive vehicle or DQ at biweekly intervals for 3 months. When mice were 23 months old, brain structure and cerebral blood flow were analyzed with MRI and postmortem histopathology (Figure 5 and Supporting Information Figure S5). Consistent with senescent cell removal, intermittent DQ treatment significantly reduced the number of NFT‐containing cortical neurons (*p* < 0.0001, 35% reduction; Figure 5a,b). Relative to the existing neuronal population at this advanced age, gene expression of the NFT‐associated senescence gene array was reduced by DQ (*p* = 0.0006; Supporting Information Figure S6a). Among these genes, those highly sensitive to NFT‐dependent upregulation (Supporting Information Figure S2) were most affected (i.e., *Tlr4: p* = 0.0459 and* Cxcl1: p* = 0.0142; Figure 5c; Supporting Information Figure S6a). NFTs are highly correlated with the rate of ventricular enlargement, an indicator of brain atrophy and hallmark of AD pathology (Silbert et al., 2003). Tau_NFT_ mice recapitulate this pathology on a wild‐type (Supporting Information Figure S1) and *Mapt^0/0^* background (*p* = 0.0007; Figure 5d). The DQ‐dependent reduction in cortical NFTs corresponded with decreased ventricular volume pathology (28% decrease, *p* = 0.05, Figure 5d,e) and a reduction in cortical brain atrophy (compared to controls: *p* = 0.0092 and *p* = 0.0274, vehicle and DQ, respectively; Supporting Information Figure S5a). The absence of a full rescue of ventricular enlargement to that of control animals was not completely unexpected considering the severity of disease and age of the animals when treatment was initiated.

Aberrant cerebral blood flow is a functional defect that occurs in AD and tau_NFT_ mice and is closely associated with cognitive impairment (Wells et al., 2015). In brain tissue with tau pathology, cerebral blood flow was elevated in tau_NFT_‐*Mapt^0/0^* vehicle‐treated mice (21% whole brain, *p* = 0.045; cortex, 48.7%, *p* = 0.051, Supporting Information Figure S5b,c) and consistent with previous reports of tau_NFT_ mice on a *Mapt^+/+^*background (Wells et al., 2015). DQ improved aberrant cerebral blood flow in tau_NFT_‐*Mapt^0/0^* mice such that cerebral blood flow was no longer statistically different from controls (Supporting Information Figure S5b,c). Overall, a composite analysis of DQ treatment in tau_NFT_ mice revealed a significant global benefit on cerebral blood flow and neurodegeneration (*p* = 0.0138; Supporting Information Figure S5d).

To elucidate whether the DQ‐dependent reduction in NFT burden, SASP expression, and improvements to brain structure and cerebral blood flow conferred neuroprotection, we measured levels of cell type‐specific protein expression in the brain. DQ‐treated mice expressed significantly higher levels of neuronal proteins (NeuN: 25%, synaptophysin: 40.8%; PSD95: 38.5%; *p* < 0.05; Figure 5f–i). The astrocyte protein GFAP was unchanged, while microglia Iba1 expression was elevated (Iba1: 40%, *p* = 0.0013; Supporting Information Figure S6b–d) suggesting that DQ‐mediated neuroprotection and decreased SASP were not derived from a reduction in pro‐inflammatory glia (astrocytes or microglia) but instead associated with fewer NFT‐containing neurons. Moreover, DQ did not alter total human tau protein levels indicating the effects were not driven by changes in tau protein expression, but rather insoluble NFTs or associated post‐translational modifications (Supporting Information Figure S7). Collectively, our data suggest that cyclic senescent cell removal of NFTs with DQ produced long‐lasting global effects on brain, as evidenced by histopathology and MRI analyses.

## 3 DISCUSSION

The inability to effectively treat tau‐associated diseases arises, in part, from a limited understanding of processes driving neurodegeneration during the prodromal period. We have identified cellular senescence, the quintessence of latent tissue degeneration, as a cellular mechanism upregulated in tau‐associated neurodegeneration. Findings in NFT‐developing transgenic mice, postmortem human AD, and PSP brain tissue support this concept.

Cellular senescence is an elaborate stress response that varies across tissues and even among cell types within tissues. Our experimental data provide an initial report of features consistent with cellular senescence in the brain (i.e., transcriptomics, upregulated *Cdkn1a* and *Cdkn2a*, SASP molecules, and altered cellular bioenergetics). A complete depiction of tau‐associated cellular senescence will require several follow‐up studies guided by these results and a close evaluation of already published data. For example, aberrant HMGB1 (Nilson et al., 2017), loss of lamin B and heterochromatin relaxation (Frost et al., 2016), and altered cellular morphology (Orr, Pitstick, Canine, Ashe, & Carlson, 2012) are all associated with tau pathology, and consistent with cellular senescence.

Tau processing is complex and requires tight regulation to maintain neuronal viability. Alterations to tau splice variant expression, post‐translational modifications (e.g., phosphorylation, acetylation, glycosylation, conformational changes, oligomerization, and cleavage), subcellular localization, etc. may result in neuronal dysfunction. As such, pathogenic tau is observed in a spectrum of neurodegenerative diseases where its histopathological characterization aids in clinical diagnoses. Our experimental results have revealed a strong association between NFT pathology and a senescence‐like phenotype. However, we cannot exclude the possibility that other tau species and/or specific post‐translational modifications may also contribute to cellular senescence. The upstream mediators driving tau‐associated cellular senescence in AD and PSP also remain unknown; however, it is tempting to speculate that tau‐induced cell cycle re‐entry may be involved (Arendt, 2012). Aberrant cell cycle re‐entry causes neuronal apoptosis and AD‐associated pathology (Park, Hallows, Chakrabarty, Davies, & Vincent, 2007) and requires soluble tau (Seward et al., 2013). The observed increase in NFT‐associated *Cdkn1a* and *Cdkn2a* gene expression may allow stressed neurons to abort cell cycle re‐entry and enter a cellular state similar to cellular senescence. In this way, NFTs formed in early pathogenic stages due to acute stress may initially protect neurons from cell death, but then contribute to neurodegeneration later in life through senescence‐like mechanisms by altering the bioenergetic state of the brain and upregulating the toxic SASP.

Pathogenic tau induces a traditional neuroinflammatory response by activating microglia and astrocytes (for recent review Laurent, Buee, & Blum, 2018). Our data suggest that NFT‐containing neurons may be active participants in perpetuating the inflammatory response as well. Future studies are required to better understand the contribution of SASP to the overall neuroinflammation phenotype common among many brain diseases. Nonetheless, our findings suggest that therapeutically targeting cellular senescence effectively interrupted a chronic neurodegenerative cascade to decrease NFT‐associated pathology and improve brain structure and aberrant cerebral blood flow even in the presence of established tau pathology in a late‐life advanced disease state. Overall, our data provide evidence that cellular senescence may be an underlying pathogenic process common among tauopathies, which opens a new field of investigative research and offers a potential druggable target to treat the >20 tau‐associated neurodegenerative diseases.

## 4 METHODS

### 4.1 Mice

All animal experiments were carried out following National Institutes of Health and University of Texas Health Science Center at San Antonio (UTHSCSA) Institutional Animal Care and Use Committee guidelines. We used 16‐ to 32‐month‐old male and female rTg4510 and rTg21221 mice that reversibly express P301L mutant human tau or wild‐type human tau 4R02, respectively, on either a wild‐type or *Mapt* knockout Bl6/FVB genetic background (Hoover et al., 2010; Santacruz et al., 2005; Wegmann et al., 2015). Nontransgene expressing littermates from rTg4510 and rTg21221 are used as controls; since no differences were found between these control lines, only littermates from rTg4510 are used here (Supporting Information Figure S1f–i). The mice were bred by Rose Pitstick and George A. Carlson at McLaughlin Research Institute, Great Falls, MT. Mouse euthanasia, brain dissection, and preparation were performed as previously described (Orr et al., 2012, 2014 ).

### 4.2 Ingenuity pathway analyses

The GEO accessions GDS2795 and GSE56772 were accessed from the GEO Profiles database (Barrett et al., 2013; Dunckley et al., 2006) with Rstudio version 1.0.143. Because the GDS2795 data set was a within‐subject design, ratios of NFT vs. CTL gene expression were generated for each subject. Mean ratios of each gene from GDS2795 and fold change values from GSE56772 were uploaded into Ingenuity Pathway Analyses (IPA) software (IPA, QIAGEN Inc., https://www.qiagenbioinformatics.com/products/ingenuity-pathway-analysis). For GDS2795, GenBank Accession IDs were used and 34,910 out of 54,675 genes were identified by IPA software. The expression fold change cutoff value was set at 3 (both down‐ and upregulated) compressing the analyses to 3,048 genes. For GSE56772, LIMMA package was used to determine the fold change and *p*‐values. The *p*‐value cutoff for IPA analysis was set at *p* < 0.01 yielding 1,294 transcripts, 738 down‐ and 556 upregulated. We utilized IPA causal analytic tools (Kramer, Green, Pollard, & Tugendreich, 2014) to elucidate predicted upstream regulators, as well as disease and biological functions with significant z‐scores enriched in our data set.

Findings from GDS2795 were replicated with more stringent criteria (*p* < 0.05 for NFT/CTL ratios with no fold change limit) allowing for 1715 genes to be uploaded into IPA with similar results. Similarly, these findings were replicated a third time using the LIMMA package, the most common method for microarray analysis. This method did not take into account within‐subject design. Using a *p* < 0.05, 1,219 differentially regulated genes were uploaded for IPA analyses; the results were similar to the original findings. Furthermore, results from GSE56772 were replicated using gene set enrichment analysis (GSEA) with default setting and similar results were obtained.

### 4.3 RNA extraction and qPCR

Frozen forebrain and cerebellum were powdered in liquid nitrogen. RNA was extracted from ~25 mg of each respective brain (or brain region) using the RNAqueous 4PCR® kit (Ambion), following the manufacturer protocol including the 15‐min DNase treatment. qPCR was performed on 25 ng RNA using the TaqMan® RNA‐to‐CT™ 1‐step kit. All gene expression analyses were made using TaqMan gene expression assays. RNA polymerase II subunit J (Polr2j) expression was used as an internal control for both mouse and human gene expression assays, Mm00448649_m1 and Hs01558819_m1, respectively. TaqMan genes expression identifiers for target genes are as follows: mouse and human *Cdkn2a*: Mm00494449_m1 and Hs00923894_m1, respectively; other mouse genes: *Cdkn1a*: Mm00432448_m1; *Glb1*: Mm01259108_m1; *Cxcl1*: Mm04207460_m1; *Tlr4*: Rn00569848_m1; *Il1β*: Mm00434228_m1; and *TNF*: Mm00443258_m1. The senescent cell population comprises a small proportion of all cells in a tissue; therefore, SASP gene expression values were normalized to neuronal *Mapt*, Mm00521988_m1; Mm00521992_m1 the senescence‐susceptible neuronal population. qPCR was performed using the Applied Biosystems 7900HT Sequence Detection System, with SDS software version 2.3. Cycle profile was performed using the kit manufacturer protocol.

### 4.4 Protein extraction and capillary electrophoresis

Approximately 50 mg frozen forebrain was used for subcellular fractionation and capillary electrophoresis as previously described (Orr et al., 2014; Orr, Garbarino, Salinas, & Buffenstein, 2015). Briefly, frozen tissue was powdered in liquid nitrogen and then homogenized with dounce and pestle and fractionated following manufacturer protocol (Subcellular Protein Fractionation Kit, Thermo Fisher Scientific). Protein concentrations were determined with BCA (Bio‐Rad); 2 µg protein was used for capillary electrophoresis. Antibodies were diluted in Wes Antibody Diluent to the final working concentrations: p65, 1:50 (Cell Signaling, D14E12; Beverly, MA); phospho‐Ser139 H2A.X, 1:50 (Cell Signaling, 20E3); HT7, 1:1,000 (Pierce/Invitrogen); NeuN, 1:50 (Millipore, MAB377; Temecula, CA, USA): GFAP 1:200 (Cell Signaling, D1F4Q); synaptophysin, 1:50 (Cell Signaling, D35E4); see Supporting Information Table S1 for complete antibody information. Protein quantification was performed by normalizing to total protein concentration (Li & Shen, 2013; Moritz, 2017; Supporting Information Figure S8).

### 4.5 Histology

Brains were fixed in 4% PFA for 48 hr and transferred to PBS containing 0.02% sodium azide and vibratome sectioned at 30 µm. Sections were washed 3× with TBS (pH 7.4) and incubated in 50% ethanol for 5 min, followed by 70% ethanol for 5 min. The sections were then submerged in 0.7% sudan black b dissolved in 70% ethanol for 5 min to quench lipofuscin‐like autofluorescence. Tissues were then rinsed three times for 1–2 min in 50% ethanol. Following this step, tissue sections were transferred from 50% ethanol to TBS and proceeded to immunofluorescence staining as described previously (Orr et al., 2015, 2014 ). Primary antibodies used are as follows: PHF1 (1:100, kind gift from Dr. Peter Davies), NeuN (1:500 Cell Signaling, D3S31), and histone 3 (1:400, Cell Signaling, D1H2; Supporting Information Table S1). Secondary antibodies are as follows: Goat anti‐Mouse IgG (H + L), Alexa Fluor 594 and Goat anti‐Rabbit IgG (H + L), and Alexa Fluor 488 (1:1,500, Thermo Fisher Scientific). Imaging was performed using a Zeiss LSM 780 confocal microscope, with ZEN 2.3 software.

### 4.6 Confocal image analyses

Image analyses were conducted using ImageJ’s FIJI. Analyses were performed on confocal z‐stacks imaged at 40x magnification. A maximum intensity image was created by compressing four z‐stack planes. All analyzed DAPI fields were applied a bandpass filter under the same conditions, applied a threshold, and measured using particle analysis excluding particles smaller than 25 µm^2^. All particles measured in the analysis were checked for mislabels, and any particles that included two nuclei or exhibited abnormal/incorrect selection were excluded from analysis. Cell type was identified using NeuN (neurons) and PHF1 (NFT‐bearing neurons) immunofluorescence.

### 4.7 SA β‐gal staining

Following euthanasia, brains were immediately removed and fresh‐frozen in an isopentane/liquid nitrogen slurry. The frozen brains were immediately adhered to the cryotome chuck with optimal cutting temperature compound (OCT) precooled to −18°C; 10‐µm coronal sections were collected and mounted on superfrost plus microscope slides (Fisher Scientific). After sectioning, slides were fixed for 10 min in 2% paraformaldehyde/0.2% glutaraldehyde at room temperature, rinsed 3× in TBS, and stained with SA β‐gal staining solution overnight (Dimri et al., 1995). Following SA β‐gal staining, sections were processed for immunofluorescence as described above.

### 4.8 Brightfield/SA β‐gal counts

SA β‐gal brightfield images were taken on a Nikon Eclipse Ci‐L microscope, with a digital site DS‐U2 camera (NIS‐Elements software BR 4.51.00). Coronally sectioned mouse brains (10 µm) were evaluated using DAPI; SA β‐gal‐positive and SA β‐gal‐negative cells in the CA2 region of the hippocampus were counted across eight tissue sections per animal (*n* = 5 per genotype). Staining was considered positive when granules of blue stain were present.

### 4.9 High‐resolution respirometry

HRR was conducted using two Oxygraph‐2k (models D and G) machines from Oroboros Instruments (Austria). To minimize mitochondrial damage associated with mitochondrial isolation techniques, we measured oxygen consumption in fresh brain tissue homogenates (Makrecka‐Kuka, Krumschnabel, & Gnaiger, 2015). Whole hippocampus, cortex, and cerebellum were homogenized with ~15 strokes using a Kontes glass homogenizer in 5% w/v ice cold MiRO6. Two milligram of brain homogenate was loaded into the chamber, and experiments were carried out when oxygen concentration in each well was saturated under atmospheric conditions (~190 nM/ml O_2_). All reagents and SUIT protocol were described previously (Pesta et al., 2011) with small modifications. Briefly, 1.25 mM ADP was sufficient for saturation in brain homogenate, rotenone was added at a concentration of 1 µM, and FCCP was added in a single injection at a concentration of 0.5 µM.

### 4.10 DQ administration

Control and tau_NFT_ *‐Mapt* ^0/0^ mice aged 19–20 months were randomized to receive DQ senolytic (5 mg/kg dasatinib [LC Laboratories, Woburn, MA] with 50 mg/kg quercetin [Sigma‐Aldrich]) or vehicle (60% Phosal 50 PG, 30% PEG 400%, and 10% ethanol) via oral gavage as described previously (Ogrodnik et al., 2017). Mice were weighed and fasted for 2 hr prior to treatment. One month after the first treatment, senolytic or vehicle gavage continued on a biweekly basis for a total of six treatment sessions over 12 weeks. Within 2 weeks of the final treatment, all mice underwent MRI analyses.

### 4.11 MRI

MRI experiments were performed on an 11.7 Tesla scanner (BioSpec, Bruker, Billerica, MA). A surface coil was used for brain imaging and a heart coil (Muir, Shen, & Duong, 2008) for arterial spin labeling. Coil‐to‐coil electromagnetic interaction was actively decoupled. Mice were maintained on 1.5% isoflurane anesthesia for MRI duration. *Anatomical MRI*: Anatomical images were obtained using a fast spin‐echo sequence with a matrix = 128 x 128, field of view (FOV) = 1.28cm × 1.28 cm, repetition time (TR) = 4,000 ms, and effective echo times = 25 ms. Thirty 1‐mm coronal images were acquired with four averages. Total scan time is equal to 8.5 min. *Cerebral blood flow (CBF)* was measured using continuous arterial spin labeling technique with single shot, spin‐echo, echo‐planar imaging and analyzed as previously described (Shen, Huang, & Duong, 2015). The images were acquired with partial Fourier (3/4) acquisition, matrix size = 64 × 64, FOV = 1.28 cm x 1.28 cm, TR = 3,000 ms, TE = 10.59 ms, and postlabeling delay = 350 ms. Seven 1‐mm coronal images were acquired with 100 repetitions. Total scan time is equal to 10 min. *Analyses:* MRI analysis was conducted using Stimulate (Center for Magnetic Resonance Research, University of Minnesota Medical School, Minneapolis, MN) running on a CentOS5 Linux Operating System (Strupp, 1996). Anatomical MRI images were used to measure cortex, subcortex, ventricle, and whole brain volume. The desired regions of interest (ROIs) were outlined, and volumes were obtained by multiplying ROI total voxels by voxel volume (0.004 mm^3^). Ventricular volume was obtained by thresholding anatomical image voxels to highlight regions of greater intensity, followed by ROI traces of the target regions. Whole brain volume was obtained by ROI trace after removal of the skull using a local Gaussian distribution 3D segmentation MATLAB code (Wang, Li, Sun, Xia, & Kao, 2009).

### 4.12 Statistical measures

#### 4.12.1 Transgenic mouse analyses

Measurements were taken from distinct samples. Key finding was repeated in separate mouse cohorts and is listed in Extended Data Figures. Each age cohort for all analyses contained three to nine animals (specified in figure legends); both males and females were included. Only females were included in DQ treatment and MRI analyses due to animal availability. Statistics were not used to predetermine sample sizes, but instead was determined empirically from previous experimental experience with similar assays and/or from sizes generally employed in the field. Data are expressed as mean ± standard error of the mean (*SEM*). Genotype and treatment comparisons were analyzed using one‐way analysis of variance (ANOVA) with Tukey’s post hoc or unpaired *t* test unless stated otherwise in the figure legends. Respirometric data, brain volume, and cerebral blood flow data were analyzed using two‐way ANOVAs (genotype × respirometric parameter) and (treatment × brain region), respectively, with Tukey’s post hoc comparisons.

#### 4.12.2 Human PSP brain tissue analyses

The PSP group contained 14 samples and was compared to 10 age‐matched controls with both sexes included; the significance was determined with unpaired two‐tailed *t* test. We performed ANOVA analysis of the log base 10 transformed CDKN2A expression as predicted by the levels of AD pathology; in addition, we used the more conservative Kendall’s Tau rank correlation to test this association as well. The human CDKN2A and AD pathology analyses were performed using the R v3+ (Vienna, Austria) environment for statistical computing using an accountable data analysis process. All other data were analyzed using GraphPad Prism version 7.0c for Mac OS X, GraphPad Software, San Diego, CA, www.graphpad.com/. Data were considered statistically different at *p* < 0.05.

## CONFLICT OF INTEREST

None declared.

## AUTHOR CONTRIBUTIONS

N.M. provided guidance and support to authors and edited the manuscript. J.M.V. designed and conducted GSEA and respirometry experiments, analyzed and interpreted data, prepared figures, wrote methods, independently confirmed key results of gene expression and histological measures, and helped write and edit the manuscript. K.R.S. managed senolytic treatment experiments, performed histological staining, conducted blinded histological analyses of immunofluorescence data, wrote methods, and edited the manuscript. E.B. assisted with senolytic treatments, conducted blinded analyses of anatomical MRI images, and wrote corresponding methods. Q.S. performed all MRI experiments and analyses, conducted blinded analyses of CBF data, and provided oversight to E.B. in anatomical analyses. C.S.T. performed histology, SA β‐gal staining, and blinded quantification, developed FIJI analysis protocols, wrote methods, and edited the manuscript. M.E.O. conceived, supervised, and attained funding for the project; designed the experiments, conducted experiments, analyzed and interpreted data, provided guidance and supervision to coauthors, prepared figures, and wrote the manuscript.

## DATA AVAILABILITY

References for source data for Figure 1 are provided with the paper; data that support Figure 1 findings are available from the corresponding author upon reasonable request. All other data supporting the findings of this study are available within the paper and its extended data files.

## References

[acel12840-bib-0001] Arendt, T. (2012). Cell cycle activation and aneuploid neurons in Alzheimer's disease. Molecular Neurobiology, 46(1), 125–135. 10.1007/s12035-012-8262-0 22528601

[acel12840-bib-0002] Arendt, T. , Rodel, L. , Gartner, U. , & Holzer, M. (1996). Expression of the cyclin‐dependent kinase inhibitor p16 in Alzheimer's disease. Neuroreport, 7(18), 3047–3049. 9116237 10.1097/00001756-199611250-00050

[acel12840-bib-0003] Arriagada, P. V. , Growdon, J. H. , Hedley‐Whyte, E. T. , & Hyman, B. T. (1992). Neurofibrillary tangles but not senile plaques parallel duration and severity of Alzheimer's disease. Neurology, 42(3 Pt 1), 631–639. 10.1212/WNL.42.3.631 1549228

[acel12840-bib-0004] Baker, D. J. , Wijshake, T. , Tchkonia, T. , LeBrasseur, N. K. , Childs, B. G. , van de Sluis, B. , … van Deursen, J. M. (2011). Clearance of p16Ink4a‐positive senescent cells delays ageing‐associated disorders. Nature, 479(7372), 232–236. 10.1038/nature10600 22048312 PMC3468323

[acel12840-bib-0005] Barrett, T. , Wilhite, S. E. , Ledoux, P. , Evangelista, C. , Kim, I. F. , Tomashevsky, M. , … Soboleva, A. (2013). NCBI GEO: Archive for functional genomics data sets–update. Nucleic Acids Researchearch, 41(D1), D991–D995. 10.1093/nar/gks1193 PMC3531084 23193258

[acel12840-bib-0006] Bennett, R. E. , Robbins, A. B. , Hu, M. , Cao, X. , Betensky, R. A. , Clark, T. , … Hyman, B. T. (2018). Tau induces blood vessel abnormalities and angiogenesis‐related gene expression in P301L transgenic mice and human Alzheimer's disease. Proceedings of the National Academy of Sciences USA, 115(6), E1289–E1298. 10.1073/pnas.1710329115 PMC5819390 29358399

[acel12840-bib-0007] Caballero, B. , Wang, Y. , Diaz, A. , Tasset, I. , Juste, Y. R. , Stiller, B. , … Cuervo, A. M. (2018). Interplay of pathogenic forms of human tau with different autophagic pathways. Aging Cell, 17(1), 10.1111/acel.12692 PMC5770880 29024336

[acel12840-bib-0008] Childs, B. G. , Baker, D. J. , Wijshake, T. , Conover, C. A. , Campisi, J. , & van Deursen, J. M. (2016). Senescent intimal foam cells are deleterious at all stages of atherosclerosis. Science, 354(6311), 472–477. 10.1126/science.aaf6659 27789842 PMC5112585

[acel12840-bib-0009] Coppe, J. P. , Patil, C. K. , Rodier, F. , Krtolica, A. , Beausejour, C. M. , Parrinello, S. , … Campisi, J. (2010). A human‐like senescence‐associated secretory phenotype is conserved in mouse cells dependent on physiological oxygen. PLoS One, 5(2), e9188 10.1371/journal.pone.0009188 20169192 PMC2820538

[acel12840-bib-0010] Correia‐Melo, C. , Marques, F. D. , Anderson, R. , Hewitt, G. , Hewitt, R. , Cole, J. , … Passos, J. F. (2016). Mitochondria are required for pro‐ageing features of the senescent phenotype. EMBO Journal, 35(7), 724–742. 10.15252/embj.201592862 26848154 PMC4818766

[acel12840-bib-0011] de Calignon, A. , Spires‐Jones, T. L. , Pitstick, R. , Carlson, G. A. , & Hyman, B. T. (2009). Tangle‐bearing neurons survive despite disruption of membrane integrity in a mouse model of tauopathy. Journal of Neuropathology and Experimental Neurology, 68(7), 757–761. 10.1097/NEN.0b013e3181a9fc66 19535996 PMC2756569

[acel12840-bib-0012] Dimri, G. P. , Lee, X. , Basile, G. , Acosta, M. , Scott, G. , Roskelley, C. , et al. (1995). A biomarker that identifies senescent human cells in culture and in aging skin in vivo. Proceedings of the National Academy of Sciences USA, 92(20), 9363–9367. 10.1073/pnas.92.20.9363 PMC40985 7568133

[acel12840-bib-0013] Dunckley, T. , Beach, T. G. , Ramsey, K. E. , Grover, A. , Mastroeni, D. , Walker, D. G. , … Stephan, D. A. (2006). Gene expression correlates of neurofibrillary tangles in Alzheimer's disease. Neurobiology of Aging, 27(10), 1359–1371. 10.1016/j.neurobiolaging.2005.08.013 16242812 PMC2259291

[acel12840-bib-0014] Flament, S. , Delacourte, A. , Verny, M. , Hauw, J. J. , & Javoy‐Agid, F. (1991). Abnormal Tau proteins in progressive supranuclear palsy. Similarities and differences with the neurofibrillary degeneration of the Alzheimer type. Acta Neuropathologica, 81(6), 591–596. 10.1007/BF00296367 1831952

[acel12840-bib-0015] Frost, B. , Bardai, F. H. , & Feany, M. B. (2016). Lamin dysfunction mediates neurodegeneration in Tauopathies. Current Biology, 26(1), 129–136. 10.1016/j.cub.2015.11.039 26725200 PMC4713335

[acel12840-bib-0016] Herbig, U. , Ferreira, M. , Condel, L. , Carey, D. , & Sedivy, J. M. (2006). Cellular senescence in aging primates. Science, 311(5765), 1257 10.1126/science.1122446 16456035

[acel12840-bib-0017] Hoover, B. R. , Reed, M. N. , Su, J. , Penrod, R. D. , Kotilinek, L. A. , Grant, M. K. , … Liao, D. (2010). Tau mislocalization to dendritic spines mediates synaptic dysfunction independently of neurodegeneration. Neuron, 68(6), 1067–1081. 10.1016/j.neuron.2010.11.030 21172610 PMC3026458

[acel12840-bib-0018] Hutter, E. , Renner, K. , Pfister, G. , Stockl, P. , Jansen‐Durr, P. , & Gnaiger, E. (2004). Senescence‐associated changes in respiration and oxidative phosphorylation in primary human fibroblasts. Biochemical Journal, 380(Pt 3), 919–928. 10.1042/BJ20040095 15018610 PMC1224220

[acel12840-bib-0019] Jurk, D. , Wang, C. , Miwa, S. , Maddick, M. , Korolchuk, V. , Tsolou, A. , … von Zglinicki, T. (2012). Postmitotic neurons develop a p21‐dependent senescence‐like phenotype driven by a DNA damage response. Aging Cell, 11(6), 996–1004. 10.1111/j.1474-9726.2012.00870.x 22882466 PMC3533793

[acel12840-bib-0020] Kirkland, J. L. , Tchkonia, T. , Zhu, Y. , Niedernhofer, L. J. , & Robbins, P. D. (2017). The clinical potential of senolytic drugs. Journal of the American Geriatrics Society, 65(10), 2297–2301. 10.1111/jgs.14969 28869295 PMC5641223

[acel12840-bib-0021] Kramer, A. , Green, J. , Pollard, J. Jr , & Tugendreich, S. (2014). Causal analysis approaches in Ingenuity Pathway Analysis. Bioinformatics, 30(4), 523–530. 10.1093/bioinformatics/btt703 24336805 PMC3928520

[acel12840-bib-0022] Laurent, C. , Buee, L. , & Blum, D. (2018). Tau and neuroinflammation: What impact for Alzheimer's disease and Tauopathies? Biomed J, 41(1), 21–33. 10.1016/j.bj.2018.01.003 29673549 PMC6138617

[acel12840-bib-0023] Li, R. , & Shen, Y. (2013). An old method facing a new challenge: Re‐visiting housekeeping proteins as internal reference control for neuroscience research. Life Sciences, 92(13), 747–751. 10.1016/j.lfs.2013.02.014 23454168 PMC3614345

[acel12840-bib-0024] Makrecka‐Kuka, M. , Krumschnabel, G. , & Gnaiger, E. (2015). High‐resolution respirometry for simultaneous measurement of oxygen and hydrogen peroxide fluxes in permeabilized cells, tissue homogenate and isolated mitochondria. Biomolecules, 5(3), 1319–1338. 10.3390/biom5031319 26131977 PMC4598754

[acel12840-bib-0025] Moritz, C. P. (2017). Tubulin or not tubulin: Heading toward total protein staining as loading control in Western Blots. Proteomics, 17(20), 1600189–1600200. 10.1002/pmic.201600189 28941183

[acel12840-bib-0026] Morsch, R. , Simon, W. , & Coleman, P. D. (1999). Neurons may live for decades with neurofibrillary tangles. Journal of Neuropathology and Experimental Neurology, 58(2), 188–197. 10.1097/00005072-199902000-00008 10029101

[acel12840-bib-0027] Muir, E. R. , Shen, Q. , & Duong, T. Q. (2008). Cerebral blood flow MRI in mice using the cardiac‐spin‐labeling technique. Magnetic Resonance in Medicine, 60(3), 744–748. 10.1002/mrm.21721 18727091 PMC2581653

[acel12840-bib-0028] Nilson, A. N. , English, K. C. , Gerson, J. E. , Barton Whittle, T. , Nicolas Crain, C. , Xue, J. , … Kayed, R. (2017). Tau oligomers associate with inflammation in the brain and retina of tauopathy mice and in neurodegenerative diseases. Journal of Alzheimer's Disease, 55(3), 1083–1099. 10.3233/JAD-160912 PMC5147514 27716675

[acel12840-bib-0029] Oddo, S. , Caccamo, A. , Shepherd, J. D. , Murphy, M. P. , Golde, T. E. , Kayed, R. , … LaFerla, F. M. (2003). Triple‐transgenic model of Alzheimer's disease with plaques and tangles: Intracellular Abeta and synaptic dysfunction. Neuron, 39(3), 409–421. 10.1016/S0896-6273(03)00434-3 12895417

[acel12840-bib-0030] Ogrodnik, M. , Miwa, S. , Tchkonia, T. , Tiniakos, D. , Wilson, C. L. , Lahat, A. , … Jurk, D. (2017). Cellular senescence drives age‐dependent hepatic steatosis. Nature Communications, 8, 15691 10.1038/ncomms15691 PMC5474745 28608850

[acel12840-bib-0031] Orr, M. E. , Garbarino, V. R. , Salinas, A. , & Buffenstein, R. (2015). Sustained high levels of neuroprotective, high molecular weight, phosphorylated tau in the longest‐lived rodent. Neurobiology of Aging, 36(3), 1496–1504. 10.1016/j.neurobiolaging.2014.12.004 25576082 PMC4869521

[acel12840-bib-0032] Orr, M. E. , Pitstick, R. , Canine, B. , Ashe, K. H. , & Carlson, G. A. (2012). Genotype‐specific differences between mouse CNS stem cell lines expressing frontotemporal dementia mutant or wild type human tau. PLoS One, 7(6), e39328 10.1371/journal.pone.0039328 22723997 PMC3377636

[acel12840-bib-0033] Orr, M. E. , Salinas, A. , Buffenstein, R. , & Oddo, S. (2014). Mammalian target of rapamycin hyperactivity mediates the detrimental effects of a high sucrose diet on Alzheimer's disease pathology. Neurobiology of Aging, 35(6), 1233–1242. 10.1016/j.neurobiolaging.2013.12.006 24411482 PMC3973159

[acel12840-bib-0034] Orr, M. E. , Sullivan, A. C. , & Frost, B. (2017). A brief overview of tauopathy: Causes, consequences, and therapeutic strategies. Trends in Pharmacological Sciences, 38(7), 637–648. 10.1016/j.tips.2017.03.011 28455089 PMC5476494

[acel12840-bib-0035] Park, K. H. , Hallows, J. L. , Chakrabarty, P. , Davies, P. , & Vincent, I. (2007). Conditional neuronal simian virus 40 T antigen expression induces Alzheimer‐like tau and amyloid pathology in mice. Journal of Neuroscience, 27(11), 2969–2978. 10.1523/JNEUROSCI.0186-07.2007 17360920 PMC6672567

[acel12840-bib-0036] Pesta, D. , Hoppel, F. , Macek, C. , Messner, H. , Faulhaber, M. , Kobel, C. , … Gnaiger, E. (2011). Similar qualitative and quantitative changes of mitochondrial respiration following strength and endurance training in normoxia and hypoxia in sedentary humans. American Journal of Physiology: Regulatory, Integrative and Comparative Physiology, 301(4), R1078–R1087. 10.1152/ajpregu.00285.2011 21775647

[acel12840-bib-0037] Prins, N. D. , & Scheltens, P. (2015). White matter hyperintensities, cognitive impairment and dementia: An update. Nature Reviews. Neurology, 11(3), 157–165. 10.1038/nrneurol.2015.10 25686760

[acel12840-bib-0038] Rodier, F. , Coppe, J. P. , Patil, C. K. , Hoeijmakers, W. A. , Munoz, D. P. , Raza, S. R. , … Campisi, J. (2009). Persistent DNA damage signalling triggers senescence‐associated inflammatory cytokine secretion. Nature Cell Biology, 11(8), 973–979. 10.1038/ncb1909 19597488 PMC2743561

[acel12840-bib-0039] Salminen, A. , & Kaarniranta, K. (2011). Control of p53 and NF‐kappaB signaling by WIP1 and MIF: Role in cellular senescence and organismal aging. Cellular Signalling, 23(5), 747–752. 10.1016/j.cellsig.2010.10.012 20940041

[acel12840-bib-0040] Santacruz, K. , Lewis, J. , Spires, T. , Paulson, J. , Kotilinek, L. , Ingelsson, M. , … Ashe, K. H. (2005). Tau suppression in a neurodegenerative mouse model improves memory function. Science, 309(5733), 476–481. 10.1126/science.1113694 16020737 PMC1574647

[acel12840-bib-0041] Sedelnikova, O. A. , Horikawa, I. , Zimonjic, D. B. , Popescu, N. C. , Bonner, W. M. , & Barrett, J. C. (2004). Senescing human cells and ageing mice accumulate DNA lesions with unrepairable double‐strand breaks. Nature Cell Biology, 6(2), 168–170. 10.1038/ncb1095 14755273

[acel12840-bib-0042] Severino, J. , Allen, R. G. , Balin, S. , Balin, A. , & Cristofalo, V. J. (2000). Is beta‐galactosidase staining a marker of senescence in vitro and in vivo? Experimental Cell Research, 257(1), 162–171. 10.1006/excr.2000.4875 10854064

[acel12840-bib-0043] Seward, M. E. , Swanson, E. , Norambuena, A. , Reimann, A. , Cochran, J. N. , Li, R. , … Bloom, G. S. (2013). Amyloid‐beta signals through tau to drive ectopic neuronal cell cycle re‐entry in Alzheimer's disease. Journal of Cell Science, 126(Pt 5), 1278–1286. 10.1242/jcs.1125880 23345405 PMC3635465

[acel12840-bib-0044] Shen, Q. , Huang, S. , & Duong, T. Q. (2015). Ultra‐high spatial resolution basal and evoked cerebral blood flow MRI of the rat brain. Brain Research, 1599, 126–136. 10.1016/j.brainres.2014.12.049 25557404 PMC4366194

[acel12840-bib-0045] Silbert, L. C. , Quinn, J. F. , Moore, M. M. , Corbridge, E. , Ball, M. J. , Murdoch, G. , … Kaye, J. A. (2003). Changes in premorbid brain volume predict Alzheimer's disease pathology. Neurology, 61(4), 487–492. 10.1212/01.WNL.0000079053.77227.14 12939422

[acel12840-bib-0046] Strupp , J. P. (1996). Stimulate: A GUI based fMRI analysis software package. Neuroimage, 3(3), S607.

[acel12840-bib-0047] Wang, L. , Li, C. , Sun, Q. , Xia, D. , & Kao, C. Y. (2009). Active contours driven by local and global intensity fitting energy with application to brain MR image segmentation. Computerized Medical Imaging and Graphics, 33(7), 520–531. 10.1016/j.compmedimag.2009.04.010 19482457

[acel12840-bib-0048] Wang, Y. , Martinez‐Vicente, M. , Kruger, U. , Kaushik, S. , Wong, E. , Mandelkow, E. M. , … Mandelkow, E. (2009). Tau fragmentation, aggregation and clearance: The dual role of lysosomal processing. Human Molecular Genetics, 18(21), 4153–4170. 10.1093/hmg/ddp367 19654187 PMC2758146

[acel12840-bib-0049] Wegmann, S. , Maury, E. A. , Kirk, M. J. , Saqran, L. , Roe, A. , DeVos, S. L. , … Hyman, B. T. (2015). Removing endogenous tau does not prevent tau propagation yet reduces its neurotoxicity. EMBO Journal, 34(24), 3028–3041. 10.15252/embj.201592748 26538322 PMC4687785

[acel12840-bib-0050] Wells, J. A. , O'Callaghan, J. M. , Holmes, H. E. , Powell, N. M. , Johnson, R. A. , Siow, B. , … Lythgoe, M. F. (2015). In vivo imaging of tau pathology using multi‐parametric quantitative MRI. Neuroimage, 111, 369–378. 10.1016/j.neuroimage.2015.02.023 25700953 PMC4626540

[acel12840-bib-0051] Zhu, Y. , Tchkonia, T. , Pirtskhalava, T. , Gower, A. C. , Ding, H. , Giorgadze, N. , … Kirkland, J. L. (2015). The Achilles' heel of senescent cells: From transcriptome to senolytic drugs. Aging Cell, 14(4), 644–658. 10.1111/acel.12344 25754370 PMC4531078

