# Supplementary material for: Tau protein aggregation is associated with cellular senescence in the brain
Source: Aging Cell. 2018 Oct 11;17(6):e12840. doi: 10.1111/acel.12840 (PMC6260915; doi:10.1111/acel.12840)

| **Supporting Data Table 1. Primary Antibody Information** | | | |  |  |
| --- | --- | --- | --- | --- | --- |
| **Antibody** | **Supplier** | **Catalog Number** | **Clone Name** | | **Lot Number** |
| p65 | Cell Signaling | 8242 | D14E12 | | 4 |
| phospho-Ser139 H2A.X | Cell Signaling | 9718 | 20E3 | | 13 |
| HT7 | Pierce/Invitrogen | MN1000 | HT7 | | QA208737 |
| NeuN | Millipore | MAB377 | A60 | | LV1746157 |
| NeuN | Millipore | MAB377 | A60 | | 2326372 |
| GFAP | Cell Signaling | 12389 | D1F4Q | | 2 |
| PHF1 | Dr. Peter Davies | N/A | N/A | | N/A |
| NeuN | Cell Signaling | 12943 | D3S31 | | 1 |
| Histone 3 | Cell Signaling | 4499 | D1H2 | | 9 |
| Synaptophysin | Cell Signaling | 5461S | D35E4 | | 2 |
| PSD95 | Abcam | ab2723 | 6G6-1C9 | | N/A |

**Supporting Data Figure 1 | The tau_NFT_ mouse model develops neurofibrillary tangles and brain atrophy.** (a-c) Histological staining of cortical brain sections from 16-month-old (a) CTL, (b) tau_WT_ and (c) tau_NFT_ mice with antibodies against neurons (NeuN, red); neurofibrillary tangles (PHF1, green) and nuclei (DAPI, blue). Only tau_NFT_ mice develop neurofibrillary tangles at this age. Scale bar 10μm. (d) Histological comparison of CTL hemibrain (left) to tau_NFT_ mouse hemibrain (right). Images illustrate severe forebrain atrophy with ventricular enlargement in tau_NFT_ mice. (e) Brain weight measurements reflect the significant neurodegeneration in tau_NFT_ mice (n=3/group; one-way ANOVA: *P* = 0.0015, Tukey’s posthoc analysis: CTL vs. tau_WT_ = 0.0644, CTL vs. tau_NFT_ = 0.0011; tau_WT_ vs. tau_NFT_ = 0.0477. Data are graphically represented as mean ± s.e.m. (f-i) Quantitative gene expression analyses on RNA isolated from CTL non-transgene expressing littermates of tau_WT_ mice (TRE- tau_WT_: closed circles) and tau_NFT_ mice (TRE-tau_NFT_: red circles) mouse forebrain indicates statistically similar expression by unpaired two-tailed t-test. (f) *Tnfa*: *P* = 0.2256 (g) *Tlr4*: *P* = 0.5230 (h) *Il1b*: *P* = 0.2244 and (i) *Cxcl1:* *P* = 0.9808. Data are graphically represented as mean with 95% CI.

**Supporting Data Figure 2 | The tau-induced senescence-associated gene expression was dependent on high molecular weight tau.** (a) Sixteen-month-old tau_WT_ and tau_NFT_ mice express similar levels of *Mapt* gene expression. (b) Representative capillary electrophoresis immunoblot using total human tau antibody (HT7) on brain homogenates from 16-mo-old tau_WT_ and tau_NFT_ mice. (c-g) Quantitative gene expression analyses on RNA isolated from control (CTL, non-transgene expressing mice), tau_WT_ and tau_NFT_ mice. (c) Some senescence-associated genes were not changed by NFT formation (i.e., (c) *Il6* or (d) *Lmnb1*). The significant upregulation in other senescence-associated genes in tau_NFT_ but not tau_WT_ mice allowed us to identify a gene expression array dependent on NFT formation. These genes include *Tnfa, Tlr4, Il1β, Cxcl1, Cdk1a and Cdkn2a*. Gene expression was normalized to neuronal *Mapt* expression and analyzed by Two-way ANOVA. N=3/mice group. (f-g) *Cdkn1a* and *Cdkn2a* are reproducibly elevated in a separate mouse cohort. Unpaired two-tailed t-test: *Cdkn1a*: *P =* 0.0086 and (g) *Cdkn2a*: *P =* 0.0016; tau_NFT_ mice (closed bars) and CTLs (TRE-tau_NFT_: open bars); n=3/group).

**Supporting Data Figure 3 | Senescence associated beta galactosidase reactivity was positively correlated with brain mass, but not neurofibrillary tangle pathology***.* (a) Senescence associated beta galactosidase (SA β-gal) staining carried out at pH 6.0 illustrated reactivity in 1-mo-old control mouse forebrain and (b) cerebellum. (c) Quantitative gene expression analyses on RNA isolated from CTL (open bar), tau_WT_ (closed bar) and tau_NFT_ (red bar) mouse forebrain revealed significantly upregulated lysosomal hydrolase *Glb1* gene expression in tau_NFT_ mice; ANOVA, *P* = 0.0070; CTL: n=3; tau_WT_ n=3; tau_NFT_: n=4. (d) SA β-gal staining of control 18-mo-old brain; CA1 (box) and cortex (brackets). (e) Tau_NFT_ mouse brains stained with β-galactosidase developed very low reactivity at pH 6.0 but (f) positive β-galactosidase activity was observed in tau_NFT_ mice when stained at physiological pH 4. (g-h) Immunostaining with anti-NeuN revealed co-labeling of SA β-gal positive cells with neurons. (i) Five experimental cohorts of CTL, tau_WT_ and tau_NFT_ 18-mo-old mice were analyzed for SA β-gal reactivity at pH 6.0; both males and females were included. The number of CA2 cells sampled on each histological slide was plotted; (j) the total number of CA2 cells counted did not differ among genotypes. (k) The percentage of SA β-gal positive CA2 cells was significantly lower in tau_WT_ mice than controls, and tau_NFT_ mice contained significantly fewer SA β-gal positive CA2 cells than CTL and tau_WT_ mice. (Repeated measures one-way ANOVA: *P =* 0.0049). (l) The percentage of SA β-gal positive CA2 cells was significantly correlated with brain mass (R^2^ = 0.4852, *P =* 0.0 039). Data are graphically represented as mean ± s.e.m.

**Supporting Data Figure 4 | Genetically ablating endogenous mouse tau reduced production of SASP.** Quantitative gene expression analyses on RNA isolated from tau_NFT_ mice on a *Mapt* wild type background (closed bars) and *Mapt* knockout background (hatched bars) mouse forebrain revealed a reduction in (a) *Tnfa*, *P =* 0.0047; (b) *Il1b, P =* 0.0949; (c) *Cxcl1, P =* 0.0559; and (d) *Tlr4, P =* 0.1494. N=3/group were analyzed by unpaired two-tailed t-test. Data are graphically represented as mean ± s.e.m.

**Supporting Data Figure 5 | Senolytic treatment provided modest benefits to brain structure and function in an advanced stage tauopathy mouse model.** (a) Whole brain, cortex and subcortex brain volume quantification from anatomical T2-weighted MRI of tau_NFT_ *Mapt^0/0^* mice (shown in Figure 4). Mice received senolytic treatment (dasatinib (D) and quercetin (Q), DQ, n=6) or vehicle (n=8) for three months and were compared to non-transgenic *Mapt^0/0^* mice (n=3). Data were analyzed with two-way ANOVA, Tukey’s *posthoc:* * *P* < 0.05; ** *P* < 0.005). (b) Representative MRI cerebral blood flow images. (c) MRI quantification of cerebral blood flow of whole brain, cortex and subcortical brain regions. Two-way ANOVA treatment main effect, *P* = 0.0057; Tukey’s *posthoc:* * *P* = 0.0141. (d) Composite analysis of tau_NFT_ + vehicle and tau_NFT_ + DQ MRI data analyzed by two-way ANOVA DQ treatment main effect: * *P =* 0.0138. (Tau_NFT_ + vehicle, n=8; tau_NFT_ + DQ, n=6; non-transgenic, n=3; all mice were on a *Mapt^0/0^* background).

**Supporting Data Figure 6 | Senolytic treatment decreased SASP gene expression but not astrocytes or microglia.** (a) Quantitative senescence gene expression array from hippocampus of vehicle-treated (open symbols, n=7) and DQ-treated (red closed symbols, n = 5) mice relative to neuronal *Mapt* gene expression. Two-way ANOVA: treatment main effect, ****P =* 0.0006. (b) Representative capillary electrophoresis immunoblot using antibodies against GFAP and Iba1 to quantify relative levels of astrocytes and microglia, respectively, in brain homogenates from vehicle or senolytic (dasatinib and quercetin, DQ) treated mice. (c) Densitometric normalization of GFAP and (d) Iba1 antibody immunoreactivity against total protein analyzed by unpaired two-tailed t-test. ** = 0.0013. N=6/group Data are graphically represented as mean ± s.e.m.

**Supporting Data Figure 7 | Senolytic treatment did not alter soluble tau expression.** (a) Representative capillary electrophoresis immunoblot using total human tau antibody (HT7) on brain homogenates from vehicle or senolytic (dasatinib and quercetin, DQ) treated mice. (b) Densitometric normalization of total tau immunoreactivity against total protein indicates similar protein expression between treatment groups. Vehicle, n=5; DQ, n=4. Data are graphically represented as mean with 95% CI.

**Supporting Data Figure 8 | Total protein concentration was used as the internal loading control for protein quantification.** (a) Representative capillary electrophoresis immunoblot on cytosolic (Cyt.) and nuclear (Nuc.) enriched fractions using antibodies against Lamin A/C and β-Actin. (b) Representative capillary electrophoresis immunoblot of brain homogenates from control or tau transgenic mice using antibodies against Lamin A/C and β-Actin. (c) Representative capillary electrophoresis immunoblot used to detect total protein concentration. (d) Quantification of Lamin A/C, (e) β-Actin and (f) total protein concentration. CTL: n = 3; tau_NFT_: n = 5; unpaired two-tailed t-test, Lamin A/C **** *P <* 0.0001*;*  β−Actin: ** *P* = 0.0097; Total Protein: *P* = 0.2916. (g) Representative electropherogram generated from capillary electrophoresis total protein immunoblot.

**Supporting Data Figure 1 | The tau_NFT_ mouse model develops neurofibrillary tangles and brain atrophy.**


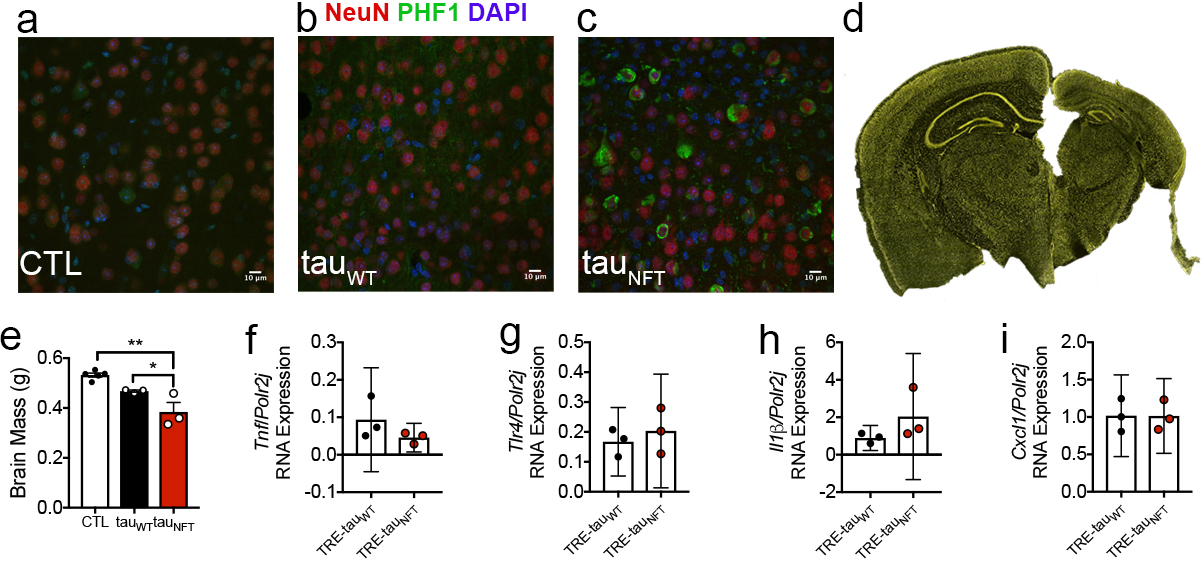


**Supporting Data Figure 2 | The tau-induced senescence-associated gene expression was dependent on high molecular weight tau.**

**
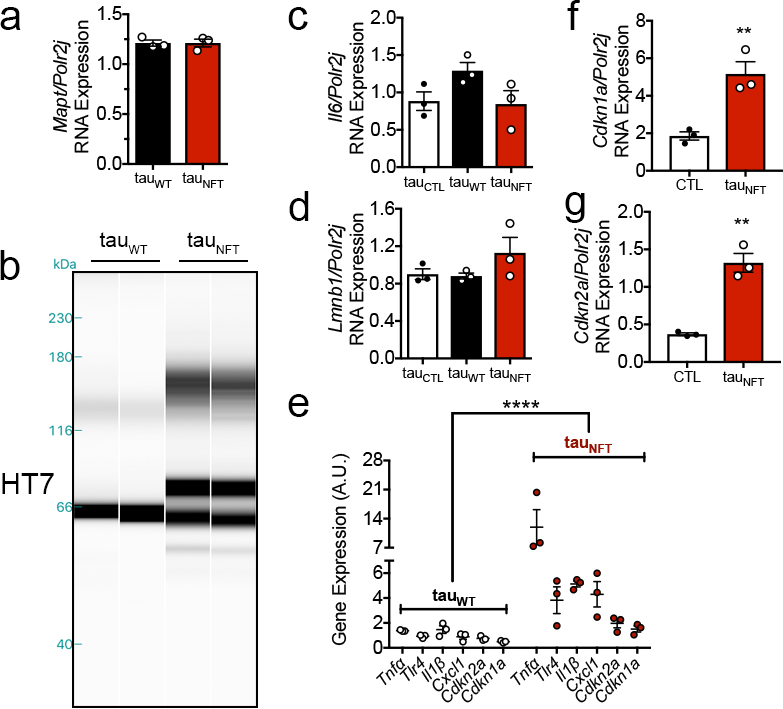
**

**Supporting Data Figure 3 | Senescence associated beta galactosidase reactivity was positively correlated with brain mass, but not neurofibrillary tangle pathology.**

**
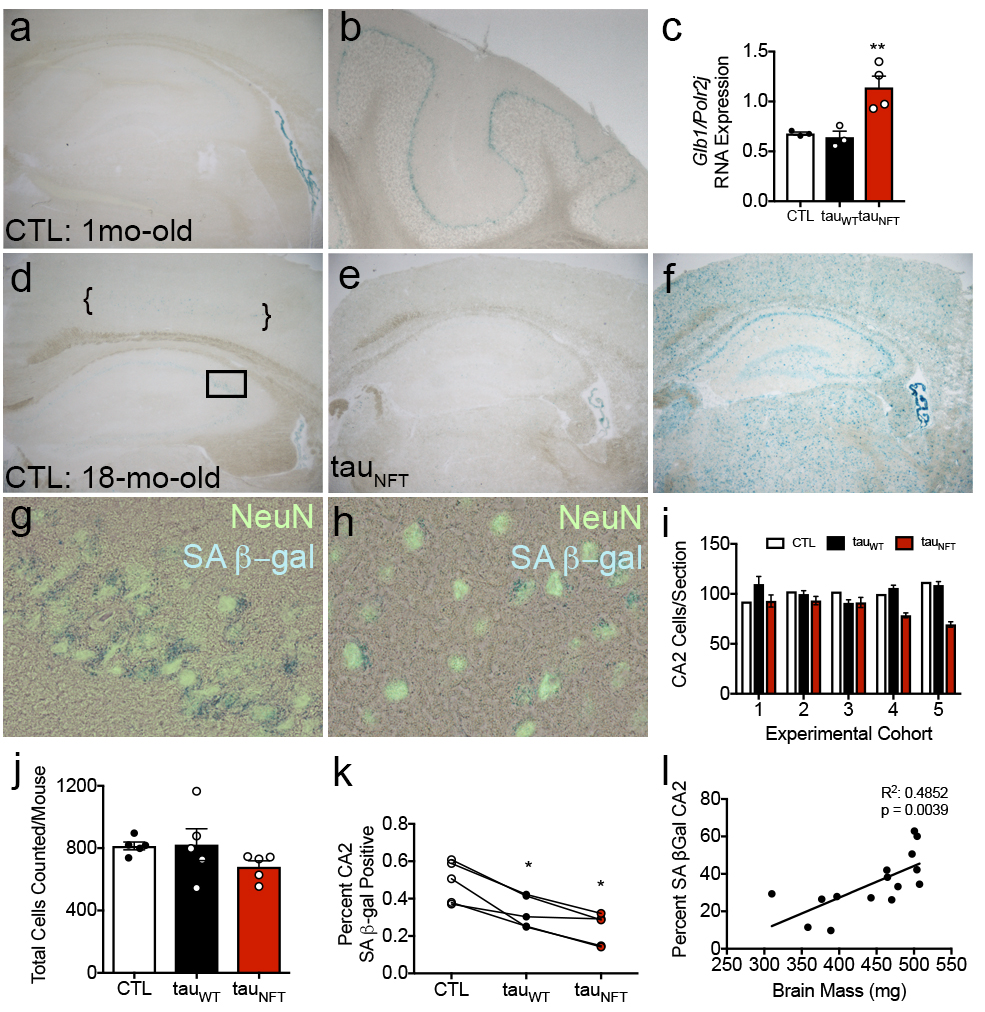
**

**Supporting Data Figure 4 | Genetically ablating endogenous mouse tau reduced production of SASP.**

**
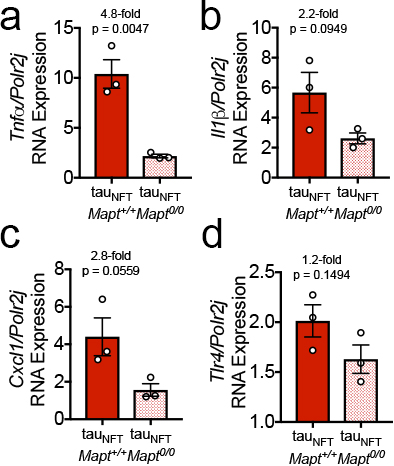
**

**Supporting Data Figure 5 | Senolytic treatment improved brain pathology.** **
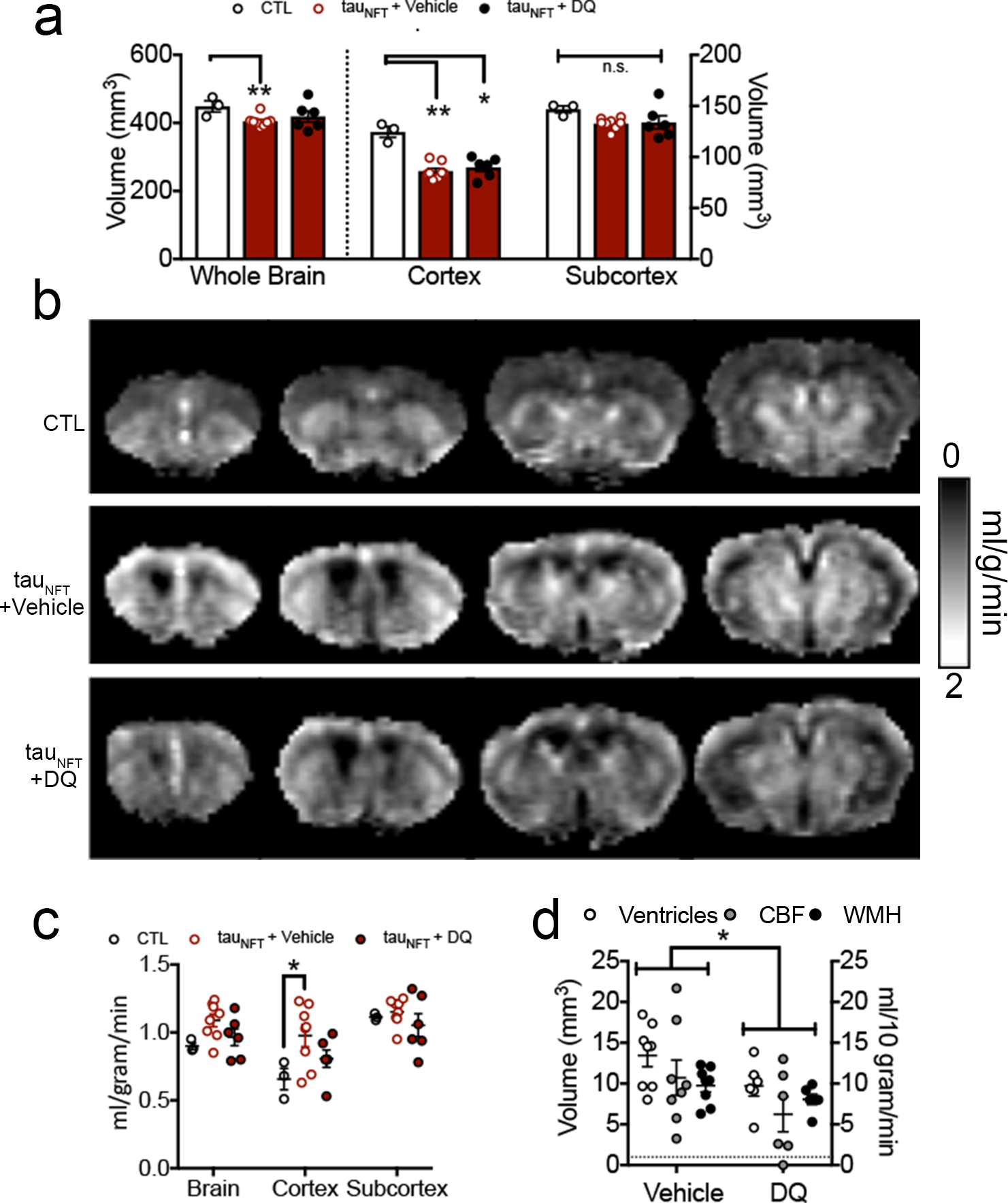
**

**Supporting Data Supporting Data Figure 6 | Senolytic treatment decreased SASP gene expression but not astrocytes or microglia.**

**
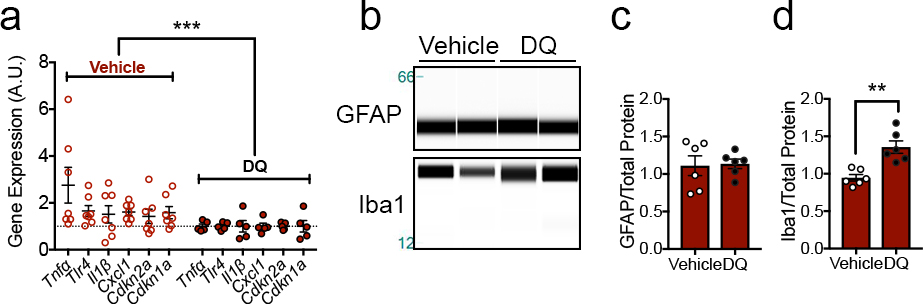
**

**Supporting Data Figure 7 | Senolytic treatment did not alter soluble tau expression.**

**
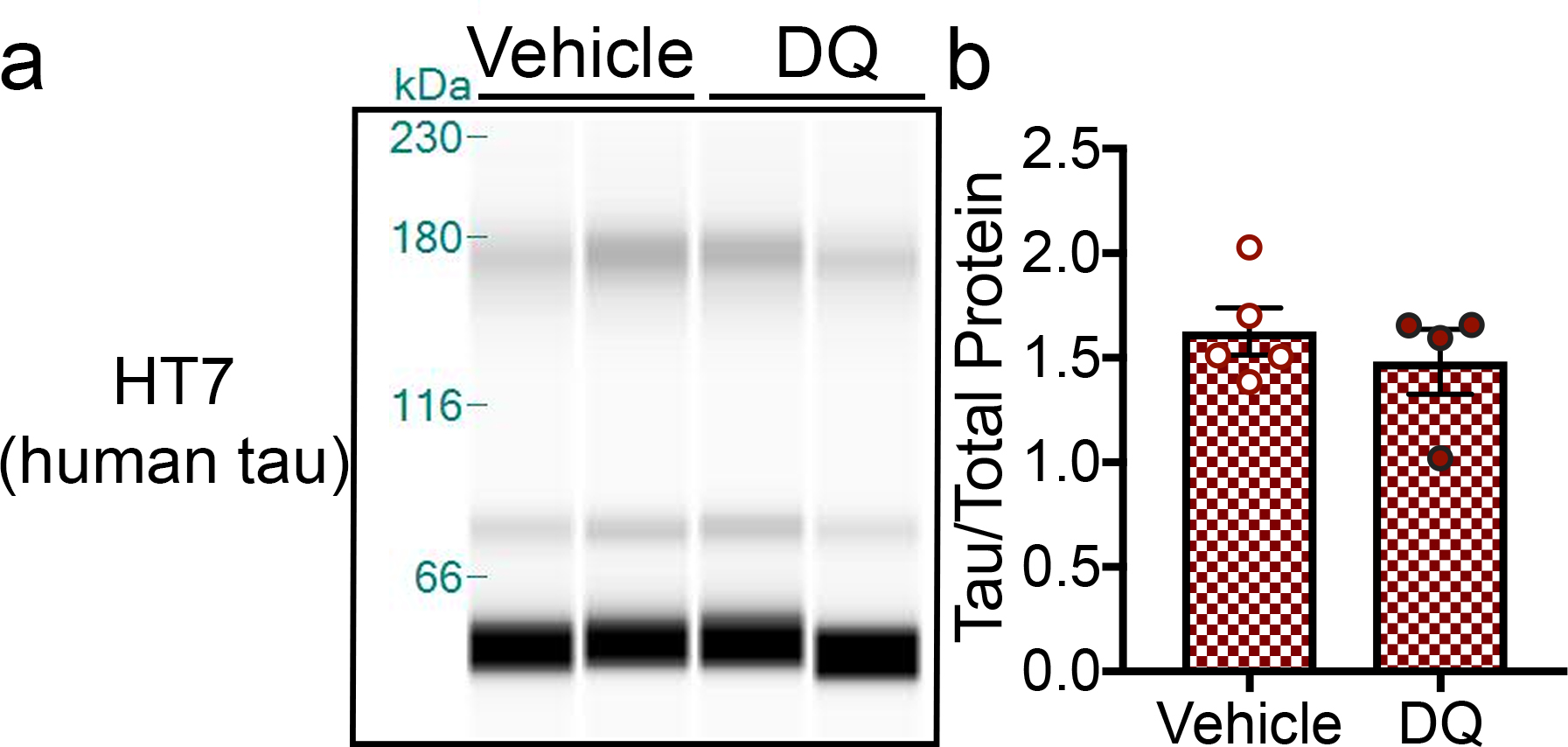
**

**Supporting Data Figure 8 | Total protein concentration was used as the internal loading control for protein quantification.**


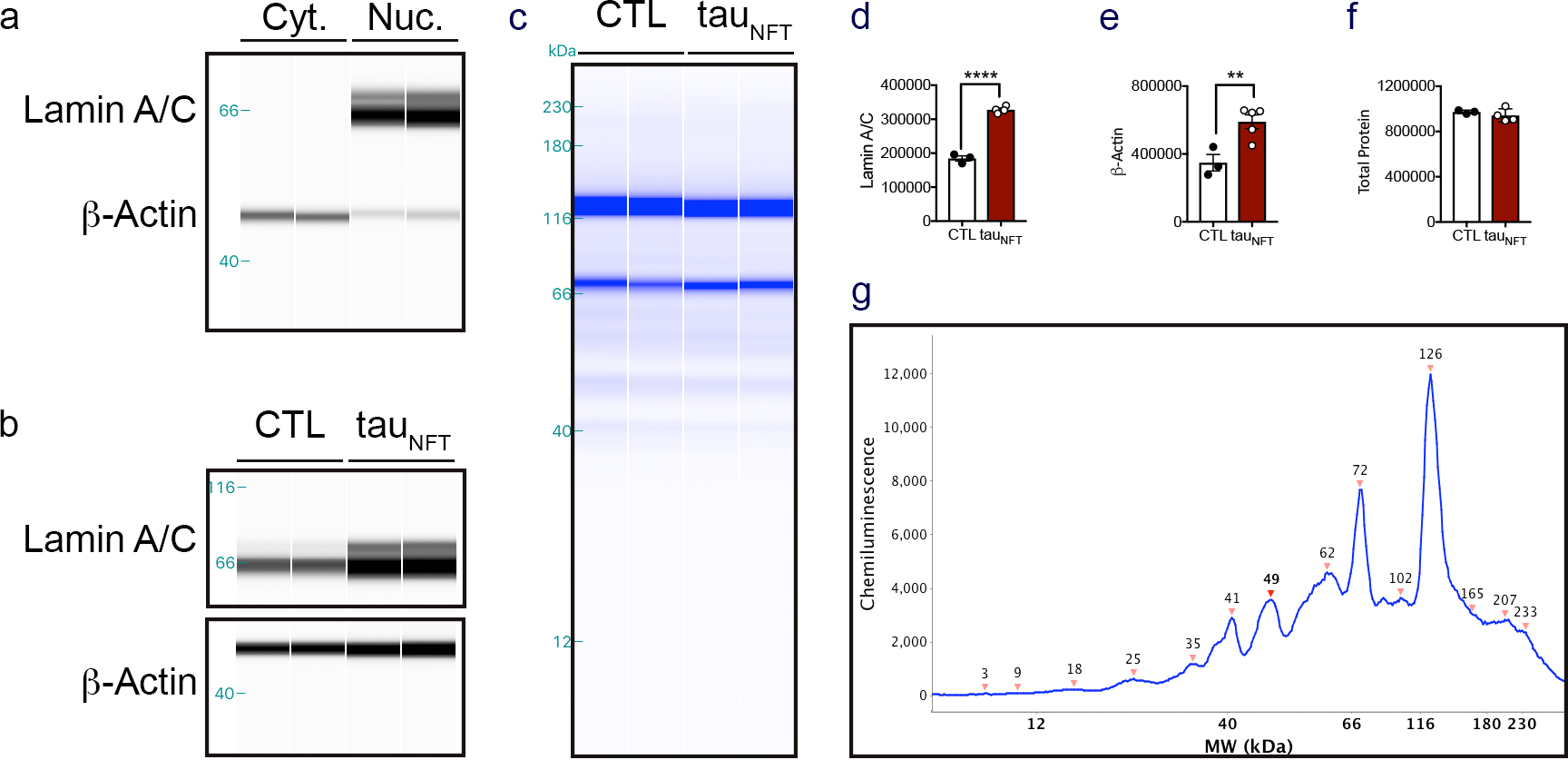

Supplement: Supplementary file 1 [file ACEL-17-e12840-s001.docx]
